# A Living Semiartificial Photoelectrocatalytic Biohybrid for Solar CO_2_ Fixation and Fermentation to Fatty Acids

**DOI:** 10.1021/acsami.5c15023

**Published:** 2025-11-10

**Authors:** Cathal Burns, Muhammed Rishan, Lee Stevens, Ellie Ashcroft, Linsey Fuller, Elizabeth A. Gibson, Shafeer Kalathil

**Affiliations:** † Faculty of Science and Environment, School of Geography and Natural Sciences, 5995 Northumbria University, Newcastle NE1 8ST, U.K.; ‡ School of Natural and Environmental Sciences, Newcastle University, Newcastle upon Tyne NE1 7RU, U.K.; § Low Carbon Energy Research and Technologies Group, Faculty of Engineering, 6123 University of Nottingham, Nottingham NG7 2TU, U.K.; ∥ Procter and Gamble Innovation Centre, Whitley Rd, Newcastle upon Tyne NE12 9TS, U.K.

**Keywords:** semiartificial photosynthesis, photoelectrochemistry, solar chemicals, biointerface, copper bismuth oxide, Sporomusa ovata, chain elongation, Clostridium kluyveri

## Abstract

To address the global climate and energy crisis, innovative strategies are urgently needed to transform CO_2_ into sustainable fuels and chemicals. We present a semiartificial biophotoelectrochemical (BPEC) platform, combining solar energy conversion with naturally evolved microbes to develop solutions for transforming CO_2_ and water into multicarbon products—without sacrificial additives or precious materials. This remains extremely challenging for fully artificial photocatalytic systems. Our system features a scalable and low-cost CuBi_2_O_4_ photocathode, stabilized by a thin MgO interlayer, in direct contact with the CO_2_-fixing bacterium *Sporomusa ovata* grown on the electrode surface. This interface enables direct electron uptake, eliminating the need for diffusible redox mediators or externally supplied H_2_—limitations commonly seen in bionic leaf systems. The BPEC operated stably for 140 h (5.5 days), a record duration for a Cu-based system, producing 673.2 ±  71.4 μM cm^–2^ acetate and 683 ± 55.2 μM cm^–2^ of ethanol with a Faradaic efficiency of 69% for C_2_ products. Subsequent addition of *Clostridium kluyveri* enabled biological chain elongation, producing 1.31  ±  0.2 μmol butyrate (C_4_) and 0.6  ±  0.1 μmol caproate (C_6_), with 0.72  ±  0.2 μmol H_2_ as a fermentation byproduct. To our knowledge, this represents the longest-chain solar-driven CO_2_-derived product reported to date, highlighting a critical advance in artificial photosynthesis. This approach demonstrates the power of pairing stable photoelectrochemical interfaces with microbial consortia to utilize CO_2_ as a feedstock for solar chemical production.

## Graphical abstract

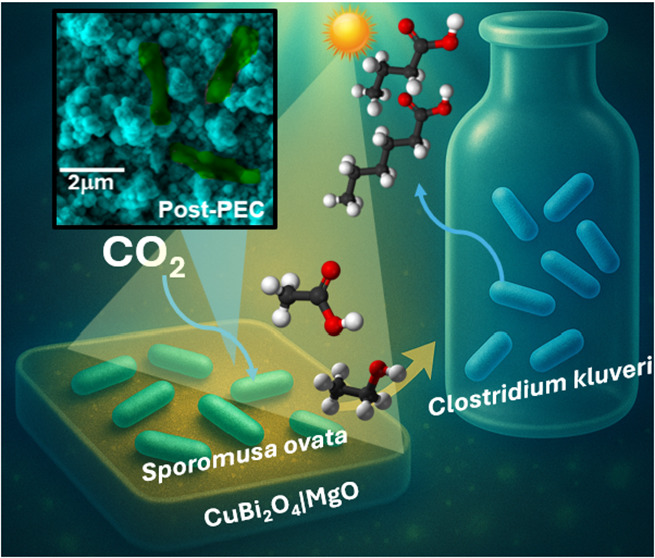

## Introduction

With the continuing global demand for energy driven by a rising population and constant industrial growth, the overreliance on fossil fuels has intensified, contributing greatly to greenhouse gas emissions, including CO_2_. As the concentration of atmospheric CO_2_ continues to rise sharply, the impacts of climate change are becoming ever-present in our lives—including global warming, severe weather events, and ocean acidification. To address this, urgent advancements in technologies that not only reduce emissions but also actively use CO_2_ as a raw material are needed. The technologies should mitigate CO_2_ emissions and repurpose the waste gas into valuable feedstocks for sustainable chemical and fuel production. This would provide a means of reducing the chemical industry’s reliance on petrochemical feedstocks.

A promising approach to address atmospheric CO_2_ levels is artificial photosynthesis, which aims to convert solar energy into valuable chemicals from earth-abundant feedstocks such as water and CO_2_. If optimized, this approach will provide a platform to offset atmospheric CO_2_ levels while producing valuable chemicals (e.g., short and medium-chain fatty acids (SCFAs/MCFAs)) remains an immense scientific challenge. Driving the formation of multicarbon products from a C_1_ feedstock using solar energy with purely artificial materials (organic or inorganic) with high efficiency and selectivity has proven to be exceptionally difficult.^1^

Of these systems, photoelectrocatalytic (PEC) systems are particularly compelling, offering the capacity to leverage solar energy as a thermodynamic driving force to drive chemical reactions. In a typical wired system, water splitting can be carried out by reducing protons to produce H_2_ with a photocathode and oxidizing water to produce O_2_ and protons with a photoanode. However, when it comes to the CO_2_ reduction reaction (CO_2_RR), purely inorganic PEC systems are still limited to producing mainly C_1_ chemicals due to the required multiple proton-coupled electron transfer (PCET) processes. For example, converting CO_2_ to ethanol (C_2_) requires 12 electron transfer steps, which is highly difficult to control on the surface of an inorganic photocatalyst alone.^2^

A promising way of overcoming the limitation of purely inorganic PEC is by integrating them with natural living catalysts (microbes) to form semiartificial biohybrids. These biohybrids benefit from the high absorption coefficients of inorganic/organic semiconductors and the selectivity of microbes, which have evolved for billions of years to metabolize CO_2_ and produce liquid organic products.^3–5^ A class of these microbes, known as electrotrophs, have evolved the ability to directly uptake electrons and use them as reducing equivalents within their metabolic pathways. Several examples are found in the literature of such semiartificial photosynthetic biohybrids for CO_2_ utilization.^6–11^ Biohybrid assemblies have been explored using both externally powered electrochemical setups with photovoltaics (PV-EC systems) and fully integrated photoelectrochemical (PEC) systems. PV-EC systems offer tunable control of the process; they have the disadvantage of requiring additional reactors, wiring, gas lines, and power-management systems, increasing the overall cost and complexity of the process. In contrast, using integrated PEC biohybrids combines the light absorption, water splitting, and CO_2_ utilization in a single platform. This minimizes energy losses, simplifies reactor design, and enables direct microbial interfacing with the semiconductor. Producing a direct interface between microbes and semiconductors is critical for understanding the fundamental electrotrophic mechanism at play. Currently, most methods in the literature depend on intermediates like H_2_ and syngas and often face challenges like material limitations of Si-based photoelectrodes in an aqueous environment, which are prone to corrosion, pH instability, and solution resistance.^12–14^ These issues underscore the necessity to study a direct PEC biohybrid system.

Colloidal photocatalysis offers a simpler method of forming semiartificial biohybrids while maintaining a direct interface between microbes and semiconductors.^15–19^ Colloidal systems, despite being simple to fabricate, require the addition of a sacrificial organic molecule (e.g., cysteine or triethanolamine), which the photocatalyst can oxidize to quench the holes generated upon light absorption; however, as the catalysis step is slow, the systems often struggle to oxidize water directly.^19,20^ The use of a sacrificial electron donor (SED) can lead to complications, especially in microbial systems. Often, the microbes can directly oxidize the SED and bypass the photocatalytic mechanism completely.^21,22^ Also, SEDs add toxicity to the microbes and additional costs to the system.^23^ This highlights the drawbacks of the current colloidal photocatalytic systems, and current research to understand the role of SEDs in greater detail is ongoing. In a sustainable system, SEDs should be avoided. To achieve this, research into developing systems based on Z-schemes^24^ and wired PEC systems is now being explored. Nocera et al. introduced a bionic leaf technology that utilized NiMoZn and CoPi water-splitting catalysts, powered by external PV cells, to evolve H_2_ that was taken up by *Ralstonia eutropha* together with CO_2_ to produce isopropanol.^14,25^ A fully integrated system using biohybrid or direct PEC approach would be simpler than using an external power source (PV panels) to drive water splitting and using H_2_ as an electron carrier in an intermediate step (i.e., H_2_ evolution followed by uptake by bacteria), leading to energy losses and inefficiencies. Wang et al.,^23,26^ described an alternative approach based on a dual light absorber photocatalyst sheet, which was capable of evolving H_2_ and O_2_ from water. *Sporomusa ovata* (*S. ovata*) was integrated onto the photosheet surface (*S. ovata*|Cr_2_O_3_/Ru-SrTiO_3_:La,Rh|ITO|RuO_2_–BiVO_4_:Mo) and produced acetate with 0.7% solar-to-acetate conversion. This approach relied on rare and expensive Ru and involved a complex design, including Cr coatings, which were found to leach into the electrolyte after 15 h, needing to be replenished to maintain the optimal efficiency.

In this article, we report the development of an earth-abundant, semiartificial biohybrid system for PEC CO_2_ conversion to C_2_ chemicals with a stable operating time exceeding 5 days. The resulting C_2_ products (acetate and ethanol) serve directly as photosynthesized feedstocks for the fermentation to produce C_4_ and C_6_ fatty acids (MCFAs). These MCFAs are increasingly recognized as valuable platform chemicals and versatile precursors for surfactants, lubricants, and specialty compounds.^27^ Currently, their production relies heavily on fossil-based petrochemical feedstocks.^27^ In contrast, our two-stage biohybrid approach offers a sustainable, carbon-neutral alternative that uses only sunlight, CO_2_, and water—potentially lowering the carbon footprint of existing industrial processes.^28–30^

Building on our earlier work, we developed a PEC system to carry out water splitting using scalable p-type metal oxides, CuO and CuBi_2_O_4_. To prevent degradation, we deposited thin, transparent, and stable layers of metal oxides (TiO_2_, NiO, and MgO).^31^ Here, we advance this concept by integrating *S. ovata*, an electrotrophic model bacterium, onto the CuBi_2_O_4_|MgO photocathode surface. This allows solar-driven direct electron transfer for CO_2_ reduction to acetate and ethanol, with water as the sole electron donor. The photosynthesized products are then used as an unaltered feedstock in a second-stage fermentation with *Clostridium kluyveri* (*C. kluyveri*), a known anaerobe capable of elongating carbon chains via reverse β-oxidation, producing MCFAs butyrate (C_4_) and caproate (C_6_).^32^ The predicted mechanism is illustrated in Figure 1.

**Figure 1:**
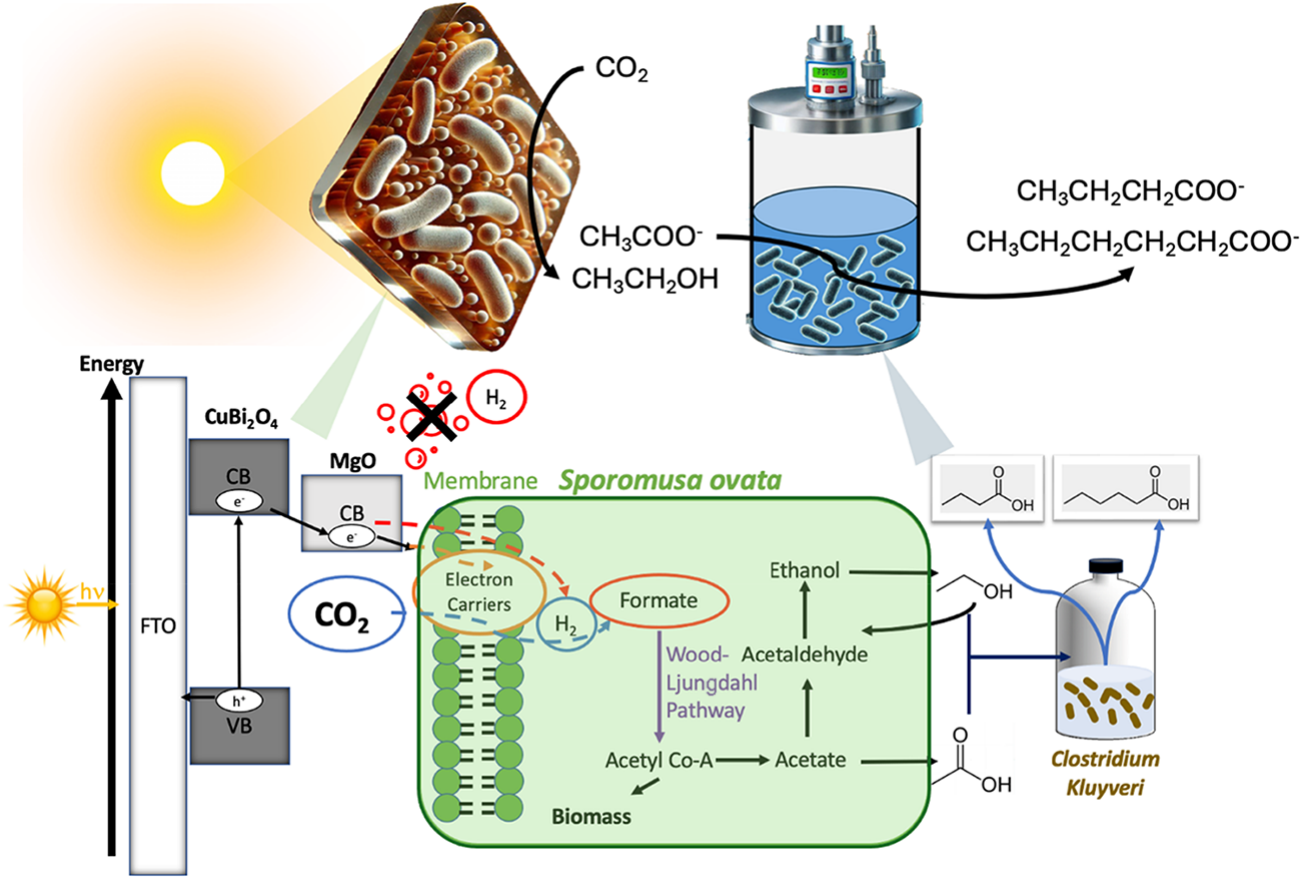
Mechanistic schematic of the semiartificial biohybrid system coupling solar-driven CO_2_ reduction to microbial chain elongation. A diagram depicting the mechanistic pathway of photosynthetic ethanol and acetate production from CO_2_ on *S. ovata*|MgO|CuBi_2_O_4_|FTO. The photocathode (CuBi_2_O_4_ layer on FTO with MgO surface passivation) absorbs visible light (hv), promoting charge separation and generating photogenerated electrons within the conduction band (CB, −0.55 V vs SHE at pH 7.0). The reduction potential of H^+^/H_2_, CO_2_/CH_3_CH_2_OH, and CO_2_/CH_3_COOH are −0.41, −0.33, and −0.27 V (vs SHE at pH 7.0), respectively. *S. ovata*, shown interacting with the surface of the photocathode, receives electrons directly via outer membrane electron carriers such as c-type cytochromes or hydrogenase (orange dashed line), or indirectly through H_2_ produced at the interface and subsequently taken up by the microbe (red dashed line). CO_2_ is taken up from the gas phase (blue dashed line) and metabolized through the Wood–Ljungdahl pathway into acetyl-CoA, forming either biomass, acetate, or undergoing further enzymatic reduction to ethanol. This ethanol and acetate are then fed to *C. kluyveri*, which utilizes the C_2_ products and elongates them into higher-value C_4_ and C_6_ carboxylic acids, specifically butyrate (CH_3_CH_2_CH_2_COO^–^) and caproate (CH_3_CH_2_CH_2_CH_2_CH_2_COO^–^), along with H_2_ gas as a byproduct.

This two-stage BPEC system establishes a direct, sustainable pathway for converting CO_2_ into industrially relevant complex organic molecules. By integrating naturally evolved microbes with sophisticated metabolic pathways, the platform facilitates highly demanding 20-electron and 32-electron transfers for the biosynthesis of butyrate and caproate, respectively. These transformations remain inaccessible to state-of-the-art synthetic catalysts, highlighting the unique power of biologically wired electrochemical systems.

## Results and Discussion

### Characterization and Assembly of the Biophotoelectrochemical (BPEC) Hybrid

CuBi_2_O_4_ was selected as a photocathode material due to its excellent visible light absorption (bandgap 1.5–1.7 eV), with the ability to evolve H_2_ from neutral water upon absorption of visible light, relative stability, and affordability.^31^ Building on our previous work, we employed a thin layer of MgO as an effective surface passivation treatment to extend the lifetime of CuBi_2_O_4_-based photocathodes.^31^ This MgO modification not only improved the photoelectrochemical performance but also provided a biocompatible interface for microbial colonization. The resulting stability of the CuBi_2_O_4_|MgO enabled successful microbial integration, forming the functional BPEC system.

We fabricated the CuBi_2_O_4_|MgO electrodes through highly accessible and scalable techniques, blade-coating for the deposition of the CuBi_2_O_4_ layer, and spin-coating of the MgO surface passivation treatment. Notably, no additional H_2_ evolution catalysts (e.g., Pt or Ru) were added to the surface. This design choice was intentional, aiming to exploit the cytochromes and hydrogenase enzymes existing within the membrane of *S. ovata* and avoiding the need for an expensive Pt or Ru catalyst, unlike previously reported systems.^23^ Moreover, this minimalist configuration allowed us to study the effect of direct electron transfer to the bacteria (via membrane-bound c-type cytochromes or even via excreted or membrane-bound hydrogenase enzymes), the mechanism for which is still not fully understood.

We first characterized the optical and electrochemical properties of the CuBi_2_O_4_ films. UV–visible absorption spectroscopy revealed strong light absorption extending up to 680 nm (Figure 2a), consistent with the material’s bandgap and confirming its suitability as a photo absorber for visible light-driven catalysis. Chopped-light linear sweep voltammetry (LSV) of the CuBi_2_O_4_|MgO displayed photocurrent densities of approximately 100 μA cm^–2^ at 0 V vs SHE (Figure 2b), indicative of effective charge separation upon illumination. Morphological analysis by scanning electron microscopy (SEM) supported by energy-dispersive X-ray spectroscopy (EDS) revealed a nanostructured film composed of spherical particles approximately 100 nm in diameter with a high abundance of Cu and Bi elements (Figures S5–S6/Table S1). Notably, the resulting porous microstructure provides a high surface area for easy access to microbial colonization, with microbes preferring to colonize porous and roughened or cracked structures as opposed to planar morphologies.^33,34^

**Figure 2:**
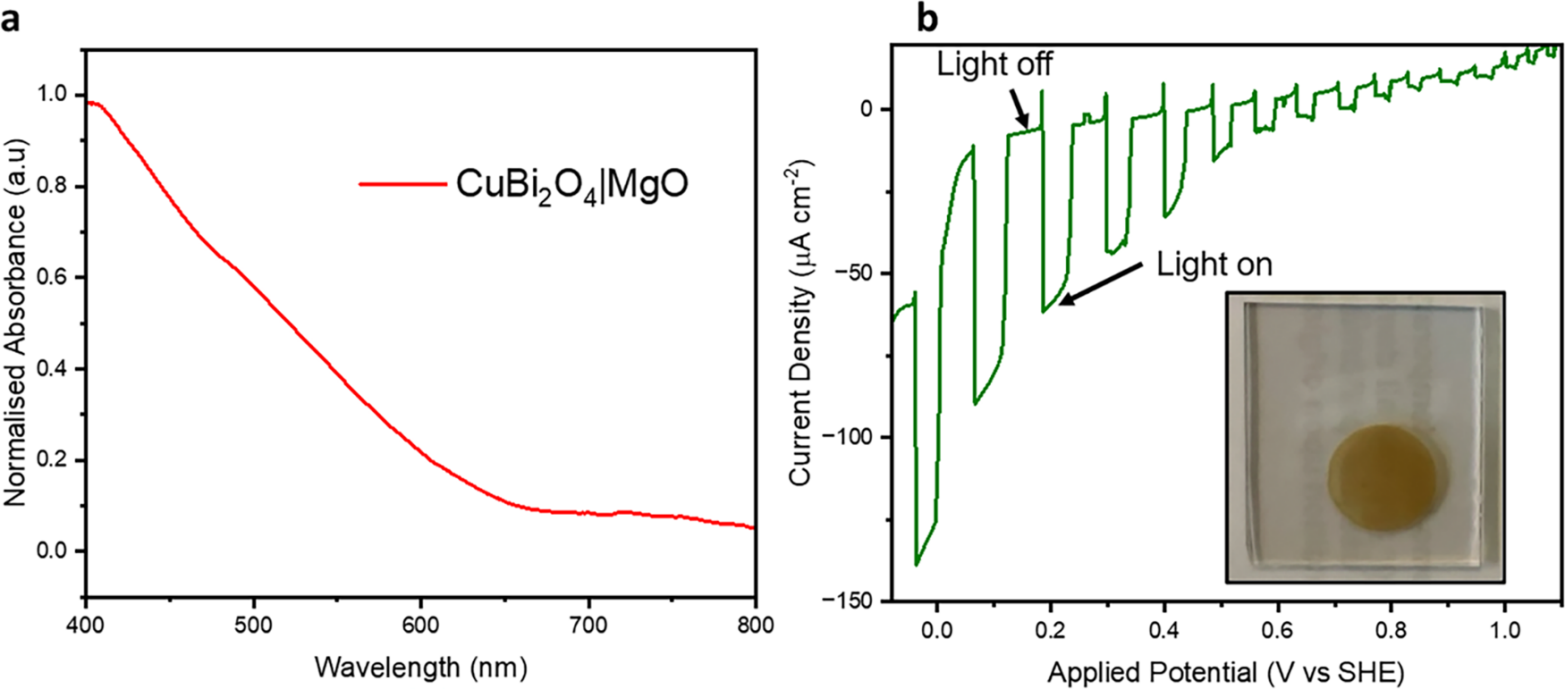
Optical and photoelectrochemical characterization of the photocathodes. (a) Visible absorption spectrum of CuBi_2_O_4_|MgO film on an FTO glass substrate. (b) Chopped-light linear sweep voltammetry (LSV) of CuBi_2_O_4_|MgO at a scan rate of 10 mV s^–1^. Inset: A photograph of a typical CuBi_2_O_4_|MgO photocathode.

Brunauer–Emmett–Teller (BET) analysis was performed to determine the specific surface area and porosity of the CuBi_2_O_4_ nanoparticle films using Krypton adsorption–desorption isotherms. The BET surface area was established to be 55.67 m^2^ g^–1^, indicating a high surface area that benefits catalysis.^35^ The BET “C” constant, associated with adsorption energy, was positive, confirming the appropriate application of the model to the isotherm. The isotherm (Figure S7a) is Type IVa,^36^ indicating a primarily mesoporous material with a mesopore volume of 0.0436 cm^3^ g^–1^, with a low amount of micropore volume of 0.0004 cm^3^ g^–1^, respectively. A characteristic hysteresis loop is observed in the multilayer relative pressure range and is associated with capillary condensation. This is supported by the Derjaguin–Broekhoff–de Boer pore size distribution^37^ (Figure S7b,c), which shows the predominance of mesoporous structures, with diameters primarily in the range of 4–10 nm. These characteristics are expected to enhance catalysis by facilitating mass transport and access to active sites. While *S. ovata* is primarily known to function in planktonic form and forms sparse or patchy biofilms, as supported by recent reports^38–40^ and evident in Figure S11, the relevance of porosity here lies in facilitating microbial–electrode interaction at the particle level. High surface area and porosity enhance the interface for direct contact between individual *S. ovata* cells and the electrode surface, thereby improving opportunities for extracellular electron transfer.

In addition to BET analysis, electrochemically active surface area (ECSA) analysis was conducted to further understand the surface of the photocathodes. Cyclic voltammetry was performed at varied scan rates (υ) (10 mV s^–1^ −500 mV s^–1^) (Figure S8a) to determine the double-layer capacitance (*C*_DL_). eq S2 shows the calculation from current density (*J*_DL_), scan rate (υ), and geometric surface area (*A*), with the *C*_DL_ calculated as 12.28 μF cm^–2^ (Figure S8b). Using eq S3, where *C*_e_ is the specific capacitance of a smooth surface (estimated at 40 μF cm^–2^ for CuBi_2_O_4_), the ECSA was calculated to be ca. 3.07 cm^2^ for an electrode of 0.5 cm^2^ geometric surface area. This data indicates that the MgO-coated CuBi_2_O_4_ nanoparticles possess a high surface area and are likely well-distributed/well-exposed to the electrolyte, properties that are particularly valuable for facilitating metabolic electron uptake by microbes. This is typical of a nanostructured film’s high porosity and roughness and correlates strongly with the BET data.

Electrochemical impedance spectroscopy (EIS) was employed to investigate the interfacial charge transfer dynamics and electrochemical behaviors of the CuBi_2_O_4_|MgO films within *S. ovata* medium as the electrolyte (Figure S9a,b). Experiments were carried out under illumination and in the dark, as well as at varied applied potentials (0.2 and 0 V vs SHE). In the dark, high charge transfer resistance (*R*_ct_) is observed due to limited free carriers, with large semicircles in Nyquist plots indicating high interfacial resistance. Illumination reduces *R*_ct_ by generating excitons, enhancing charge transfer efficiency. At 0 V vs SHE, a cathodic bias increases *R*_ct_ due to charge accumulation and bulk recombination, but illumination significantly decreases *R*_ct_, reflecting the improved carrier dynamics. The EIS data is discussed in full within the Information (Figure S9a,b).

### Biophotoelectrochemistry

To construct the biohybrid between *S. ovata* and CuBi_2_O_4_|MgO, the photoelectrodes were immersed within an adapted *S. ovata* medium with no external carbon sources or electron donors (Table S2). The CuBi_2_O_4_|MgO served as a photocathode (working electrode) in a three-electrode configuration, with a Pt wire as the counter electrode and an Ag/AgCl reference electrode. Upon illumination and application of a bias at 0 V vs SHE, photocurrent was observed, accompanied by H_2_ evolution. System purging with an 80% N_2_: 20% CO_2_ gas mixture followed by headspace analysis after 140 hours of continuous illumination revealed H_2_ production of 76  ±  12 nmol cm^–2^, corresponding to a negligible Faradaic efficiency (FE) of ∼0.4%. This low FE can be attributed to CuBi_2_O_4_ having moderate activity for H_2_ evolution, especially with the addition of an MgO passivating layer and without the addition of a cocatalyst to assist H_2_ evolution. No CO_2_RR products were observed in abiotic experiments (Table S3). The observed photocurrent in the abiotic system likely reflects background processes such as capacitive current, charging/discharging, or other nonproductive electron transfer pathways, none of which led to measurable chemical products. Crucially, this absence of appreciable H_2_ or CO_2_RR product formation under abiotic conditions provided an ideal baseline to probe the possibility of direct electron transfer from the photoelectrode to *S. ovata*.

To initiate the CO_2_RR, the same system was formed with the addition of *S. ovata* cells (OD_600_ = 0.5–0.7) in 25 mL of media (with no additional carbon source or SED) (Table S4) within the sealed reaction vessel in an 80:20 N_2_/CO_2_ atmosphere. *S. ovata* acted as a living cocatalyst for CO_2_-to-C_2_ conversion. An illustration of the proposed system is shown in Figure 3a.

**Figure 3:**
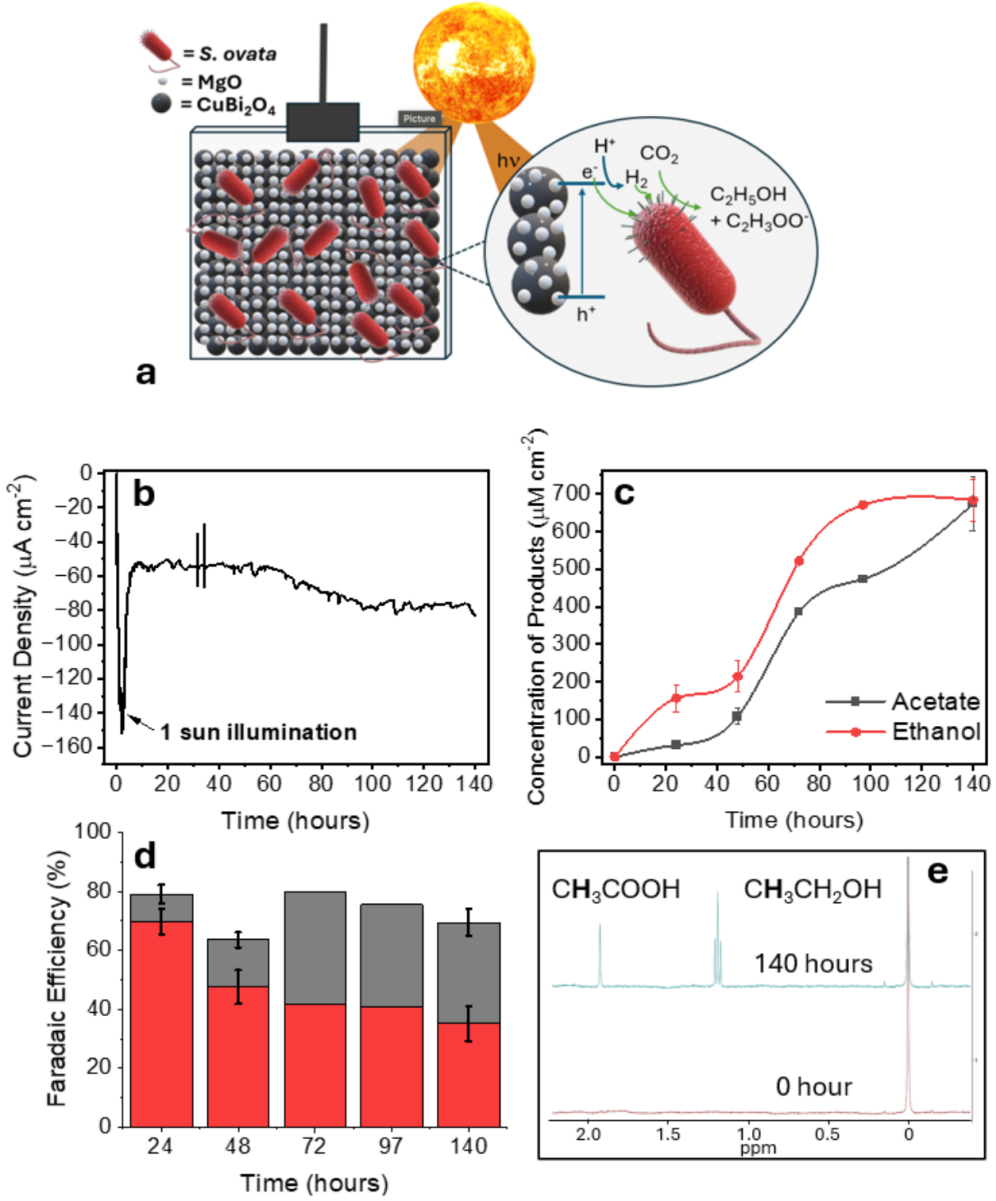
BPEC activity of the *S. ovata* | Photocathode biohybrids. (a) Illustrative diagram displaying the proposed mechanism at the biointerface. Briefly, visible light is absorbed by the CuBi_2_O_4_ (which is passivated by MgO). The photogenerated electrons are then utilized for direct electron transfer to *S. ovata* or indirect electron transfer via H_2_ evolution and H_2_ acting as a mediator. *S. ovata* utilizes these equivalents to reduce CO_2_ to C_2_ products. (b) Chronoamperometry of CuBi_2_O_4_|MgO|*S. ovata* at 0 V vs SHE under constant illumination with 1 sun (300W Xe arc lamp, AM 1.5G) for 140 h. (c) Time profile showing the accumulation of acetate and ethanol throughout the BPEC reaction. (d) Bar chart showing the Faradaic efficiencies for acetate (black) and ethanol (red) throughout the PEC reaction. (e) A typical ^1^H NMR spectrum (D_2_O, 400 MHz) before and after 140 h PEC reaction with TSP as an internal standard in D_2_O. The reactions were carried out in 25 mL reaction vessels purged with 80% N_2_: 20% CO_2_ (pH 7.0) under ambient conditions. Error bars correspond to s.d (*n* = 3 independent samples).

Chronoamperometry was performed on the illuminated CuBi_2_O_4_|MgO|*S. ovata* biohybrid system at 0 V vs SHE for 140 h (Figure 3b), marking one of the longest reported operational stabilities for a Cu­(II)-based photocathode in aqueous solution under continuous illumination. Structural stability was confirmed by X-ray diffraction (XRD) of the films before and after PEC, showing no discernible changes in the diffractograms postcatalysis (Figure S10 **)**. This remarkable durability is attributed to the MgO passivation, which inhibits surface photocorrosion and stabilizes interfacial charge transfer processes.^31,38^

Upon illumination, the chronoamperometry in Figure 3b shows an initial spike to ca. −150 μA cm^–2^, which stabilized between −60 and −80 μA cm^–2^ for the 140 h reaction period. The observed product formation during the biohybrid PEC experiments can be explained by examining the underlying metabolic pathway and electron flux dynamics in *S. ovata* in the conversion of CO_2_ to ethanol and acetate, particularly via the Wood-Ljungdahl pathway and the subsequent reduction reactions.^39,40^ ^1^H NMR was carried out throughout the BPEC reaction to monitor product formation. Under standard growth conditions, with betaine as the electron donor, acetate is the dominant product (Figure S1). However, in the BPEC system—lacking any exogenous carbon source or SED, in addition to acetate, ethanol was also produced at a comparable rate (Figure 3c). This observation suggests that electron transfer to *S. ovata* likely plays a crucial role in favoring ethanol production, providing electrons as reducing equivalents of sufficient energy to directly produce ethanol (a reduced intermediate that can later undergo oxidation to acetaldehyde and acetate, rather than being the terminal metabolite). As the reaction progressed, the observed FE’s for both C_2_ products (Figure 3d) showed an initial preference for ethanol, then by 72 h, the selectivity of both C_2_ products was very similar (ca. 41 and 38% for ethanol and acetate, respectively). This is unsurprising when looking into the metabolic pathway of *S. ovata*. In a system with excess reducing power or electron donors, acetate becomes the dominant product.^41^ By the end of the BPEC reaction (at 140 h), the selectivity had shifted to an overall FE_Ethanol_ = 35% and FE_Acetate_ = 34% (Figure 3d), indicating a gradual progression toward acetate dominance. After 140 h, the concentration of acetate and ethanol were 673.2 ± 71.4 μM cm^–2^ and 683.1 ± 55.2 μM cm^–2^, respectively. The overall FE for CO_2_-to-C_2_ products was ca. 69% after 140 h (Table S4). This leaves approximately 30% of the current that the microbes can use to sustain life.^42^

The Zeta-potential of *S. ovata* cells was measured to be −47.4 mV, while that of CuBi_2_O_4_ particles was −26.9 mV. These results indicate that both surfaces carry net negative charge, making direct electrostatic attraction unlikely to play a dominant role in the cell-photocathode interaction. It should be noted that in the PEC cell, the CuBi_2_O_4_ particles are immobilized as a film under illumination and an electrochemical bias. The PEC system contains a multitude of ions from the adapted *S. ovata* medium (electrolyte), which can interact with either the surface of the cell or photocathode (or both), potentially screening charges and rendering electrostatic interactions more favorable. Our results, together with previous reports,^23^ demonstrate that *S. ovata* cells indeed localize on the surface of metal oxides under illumination. Nevertheless, the exact mechanism governing the attachment remains unresolved and is a fundamental open question within the field.

Crucially, the electron transfer mechanism from the photocathode to *S. ovata* in this system likely involves a combination of pathways. The extremely low H_2_ evolution (∼76  ±  12 nmol cm^–2^; < 0.4% Faradaic efficiency) and the absence of abiotic CO_2_RR products suggest that indirect electron transfer via H_2_ is minimal. However, given that *S. ovata* possesses uptake hydrogenases, we acknowledge that complete exclusion of this pathway is not possible. Nonetheless, the sustained production of acetate and ethanol over the 140 h period strongly supports the hypothesis that electrons are transferred directly from the photoelectrode surface to membrane-bound redox-active components in *S. ovata*, such as c-type cytochromes or hydrogenases. While we did not perform genetic knockouts or inhibitor studies in this work, our results align with prior reports of *S. ovata*’s electrotrophic behavior on electrodes under anaerobic conditions.^43^ Future studies using redox mediators, mutants, or in situ spectroelectrochemistry would help distinguish the relative contribution of each pathway. The pH was maintained between 6.8 and 7.2 throughout all experiments, and no significant change was observed post-BPEC. Systematic deletional control experiments were conducted by omitting illumination and using heat-killed cells. No CO_2_RR products were detected via NMR for these experiments (Table S3). An additional control experiment was carried out to confirm that acetate production was not coming from ethanol oxidation. This was confirmed by running an abiotic experiment with *S. ovata* medium, 1 mM ethanol, purged with N_2_/CO_2_, CuBi_2_O_4_|MgO as the working electrode, and no *S. ovata* cells present. The results showed that after 48 h of reaction, no ethanol had been oxidized and therefore no acetate had been produced.

Post-BPEC analysis via SEM and SEM-EDS (Figure S11) revealed the physical presence of *S. ovata* cells dispersed on the photoelectrode surface, without significant alterations to the morphology of the CuBi_2_O_4_|MgO architecture. This is consistent with the lack of changes in crystal structure observed in the XRD pattern pre- and post-BPEC (Figure S10). The observed electrode characteristics—high surface area, accessible mesoporosity, and morphological stability—are critical to enabling efficient, stable, and long-term microbial–semiconductor interaction under BPEC conditions, regardless of the microbial mode of attachment.

An additional key advantage of our system is its operation without a proton exchange membrane (separating the anode and cathode), which is costly and often considered one of the largest bottlenecks to overcome before an economically feasible scale-up can be considered.^44,45^ However, *S. ovata* is not as oxygen-sensitive as previously assumed.^7^ To evaluate the potential impact of oxygen evolution at the counter electrode in our membrane-free setup, we estimated the theoretical O_2_ accumulation resulting from water oxidation. Assuming 100% FE for O_2_ evolution during 140 h of PEC operation and using the total charge passed (derived from the chronoamperometry in Figure 3b), we calculate a maximum of ∼39 μmol of O_2_ produced. Given the reactor headspace volume (∼25 mL), this corresponds to an O_2_ concentration of approximately 3.84% v/v. This is below the reported tolerance threshold of *S. ovata*, which has been shown to survive and remain metabolically active at O_2_ levels over 4% v/v.^22^ Genomic analyses support this observation, revealing the presence of oxygen-handling proteins such as rubredoxins, flavodoxins, and superoxide dismutase. These constituents allow *S. ovata* to survive in low O_2_ environments.^46^

Further confirmation of the system’s biocompatibility was obtained by confocal laser scanning microscopy (CLSM), which was utilized in tandem with a LIVE/DEAD viability assay to determine cell viability within the biofilm post 140 h of BPEC operation on the CuBi_2_O_4_|MgO surface (Figures S11–S12). The live/dead assay used two fluorescent stains: SYTO 9, which labels both live and dead cells, and propidium iodide (PI), which selectively stains cells with compromised membranes (red fluorescence), indicating cell death. CLSM imaging revealed a considerable population of cells forming a biointerface at the CuBi_2_O_4_|MgO, with 87% of the cells confirmed to be alive (calculated from the ratio of red: green fluorescence). 13% of the total cells displayed red fluorescence, meaning that cell death was minimal (Figure S12), suggesting that photoelectrons/PEC-generated H_2_ at the biointerface was sufficient to maintain electrotrophic/hydrogenotrophic microbial life. This result, combined with XRD analysis of the photocathode post-BPEC, demonstrates the long-term stability and functional robustness of the CuBi_2_O_4_|MgO|*S. ovata* system.

X-ray photoelectron spectroscopy (XPS) analysis was performed on CuBi_2_O_4_ and CuBi_2_O_4_|MgO electrodes before and after PEC operation to investigate the chemical state of the surface of the photocathodes (Figure S13). For the CuBi_2_O_4_ electrode, the survey and high-resolution spectra reveal well-defined Cu 2p peaks with characteristic satellite peaks, which are consistent with Cu^2+^ in the spinel structure. The Bi 4f doublet at ∼159 and ∼165 eV confirmed the presence of Bi^3+^, while the O 1s signal centered between 529 and 531 eV corresponds to lattice-bound oxygen with minor contributions from hydroxyl species.

Upon incorporation of MgO onto the CuBi_2_O_4_ surface, the XPS spectra show additional contributions from Mg 2p at ∼50 eV, confirming the successful surface addition. In comparison to the bare CuBi_2_O_4_ surface, the O 1s peak is broader and slightly shifted upon the addition of MgO, indicative of the coexistence of multiple oxygen environments, and these include Mg–O bonds and possibly surface hydroxyls. The Cu and Bi peaks remain characteristic of Cu^2+^ and Bi^3+^, as before. This suggests that MgO incorporation does not disrupt the bulk electronic states of CuBi_2_O_4_, but MgO does alter the surface chemistry.

XPS after PEC operation revealed notable changes to the CuBi_2_O_4_|MgO photocathode. The Cu 2p signal intensity decreases, and the satellite features are less pronounced, suggesting slight reductions of Cu^2+^ on the electrode surface under PEC conditions. The Bi 4f doublet remains, albeit with reduced intensity too. The Mg 2p contribution is still detectable, though with signs of surface hydroxylation. The O 1s spectrum shifts toward higher binding energies (532 eV) and is significantly broader, consistent with increased surface hydroxylation and small surface defects forming, induced by PEC operation.

Raman spectroscopy was employed to probe the interaction between *S. ovata* and CuBi_2_O_4_-based material postcatalysis, revealing distinct spectral modifications indicative of biohybrid formation (Figure S14a,b). The pristine CuBi_2_O_4_ spectrum exhibited characteristic vibrational modes associated with Cu–O and Bi–O bonds, which were significantly attenuated upon incorporation of MgO and exposure to electrolyte, suggesting surface interactions or phase modifications. Upon introduction of *S. ovata*, a pronounced increase in Raman intensity was observed, particularly at higher wavenumbers (>1000 cm^–1^). This can be attributed to biomolecular vibrations from proteins, lipids, and polysaccharides, confirming the successful integration of microbial components with the inorganic catalyst, as well as some scattering effects observed from the biomass on the electrode surface. Similarly, a control experiment was conducted on a glass substrate functionalized with *S. ovata*. *The Raman spectra of these samples* displayed enhanced vibrational features across the fingerprint region (500–1500 cm^–1^) and in the 2800–3500 cm^–1^ range, corresponding to C–H and O–H stretching modes, indicative of microbial biomass. Both spectra featuring *S. ovata* contain a clear band at 2900–3100 cm^–1^, typical of C–H stretching vibrations from both CH_2_ and CH_3_ groups, which is consistent with the presence of organic material (*S. ovata*). These spectra collectively support a strong interaction between *S. ovata* and CuBi_2_O_4_, highlighting the presence of cells on the electrode surface post-BPEC.

The role of the MgO interlayer within our BPEC system was primarily to stabilize the CuBi_2_O_4_ by passivating the surface and preventing photocorrosion. The mechanisms behind the MgO layer were studied in more depth using chopped-light linear sweep voltammograms and Raman spectroscopy (Figure S15a–c). In this, bare CuBi_2_O_4_ exhibits large photocurrent spikes upon light cycling on/off and gradual current decay, which is consistent with a large proportion of surface recombination and photocorrosion. It is likely that a substantial quantity of this photocurrent corresponds to Cu^2+^ reduction as opposed to H^+^ reduction. In contrast, upon the addition of MgO to the CuBi_2_O_4_ surface, we have observed stable and flat photocurrent profiles with suppressed photocurrent spikes across all light on/off cycles. This behavior is indicative of MgO preventing photocorrosion and acting as a conformal physical barrier that passivates surface defect states and reduces recombination. Additionally, a more detailed analysis of Raman spectroscopy (Figure S15c) was used to further understand the mechanism at the CuBi_2_O_4_|MgO interface. Throughout the spectra, a suppression and broadening of CuBi_2_O_4_ vibrational modes, combined with slight peak shifts upon the addition of MgO, were observed. These changes are consistent with interfacial strain and defect reduction induced by MgO.

Together, these results show that MgO stabilizes CuBi_2_O_4_ through physical passivation, interface modification, and is actively shown to reduce recombination pathways and defect states at the CuBi_2_O_4_ surface. In the future, the system can be improved by reducing the observed “blocking” effects by using more precise deposition techniques, e.g., atomic layer deposition (ALD), which could achieve nanometer control of MgO and facilitate quantum effects like tunnelling that will facilitate more efficient electron transfer to the catalyst at the electrolyte interface.

### Chain Elongation of Photosynthesized Acetate and Ethanol

Acetate and ethanol produced by the BPEC system serve as versatile carbon feedstocks for industrial applications. Acetate, for example, can be used as fuel in microbial fuel cells that use electrotrophic bacteria such as *Geobacter sulfurreducens*, which oxidize acetate to generate electricity on electrode surfaces.^23^ Additionally, the solar-produced ethanol and acetate can be directly repurposed—without any additional nutrients or fresh media—as fermentation substrates for *C. kluyveri* to produce MCFAs (Figure 4). MCFAs such as butyrate and caproate are valuable platform chemicals with widespread uses in industries, including agriculture, food, pharmaceuticals, and bioenergy. The overall stoichiometry of the chain elongation reaction is summarized in eq 1, reflecting the metabolic conversion of solar-derived C_2_ compounds into longer-chain, energy-dense fatty acids.

1 $$6C_{2}H_{5}\mathrm{OH}+3C_{2}H_{3}\mathrm{CO}O^{-}\to {C_{6}H_{11}\mathrm{CO}O^{-}+3C_{4}H_{7}\mathrm{CO}O^{-}+H}^{+}+{2H}_{2}+4H_{2}O$$

**Figure 4:**
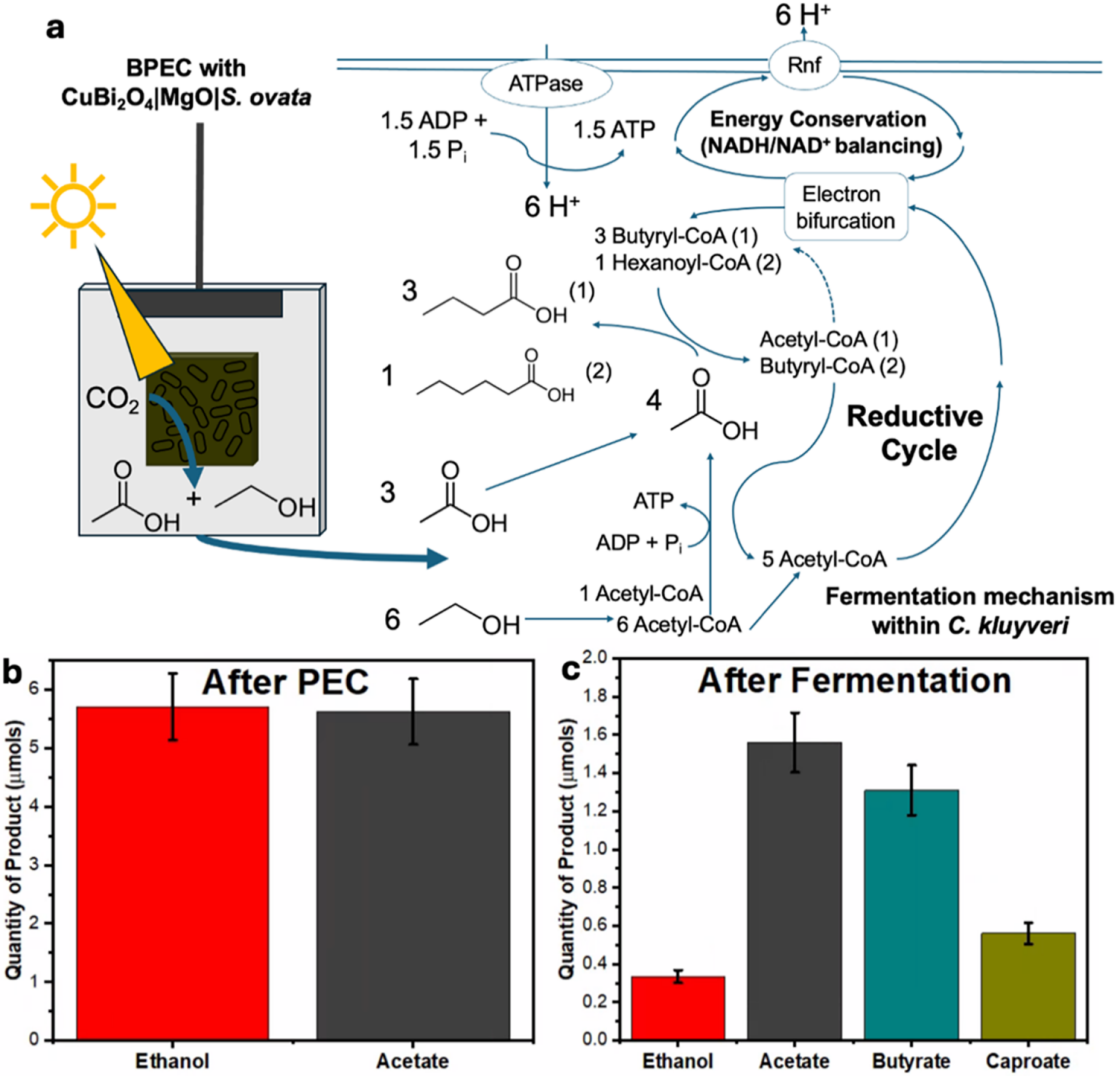
(a) Schematic representation of BPEC-driven CO_2_RR to C_2_ chemicals (acetate and ethanol), coupled to the fermentation mechanism with *C. kluyveri*. Briefly, the reverse β-oxidation cycle converts ethanol to acetyl-CoA, with one molecule in every six being oxidized to produce an additional acetate equivalent for utilization in adenosine triphosphate (ATP) production. The remaining 5 acetyl-CoA are combined with acetyl-CoA generated in the reverse β-oxidation cycle, with the first cycle producing 3 equiv of butyryl-CoA. This is then converted to butyrate or cycled again and combined with an additional acetyl-CoA molecule, yielding hexanoyl-CoA, which can yield caproate. Alongside, an energy regeneration mechanism is cycled as protons are pumped across the cell membrane, which balances the NADH/NAD^+^ pool. This proton pool creates a force that allows enhanced recovery of 2.5 ATP molecules per cycle. (b) Quantities of ethanol and acetate produced via the BPEC reaction with *S. ovata*. (c) Quantities of acetate, ethanol, butyrate, and caproate detected postfermentation (3 days) with *C. kluyveri*. Error bars correspond to s.d (*n* = 3 independent samples).

An initial control experiment was conducted to observe butyrate and caproate production in a culture with externally added ethanol and acetate using *C. kluyveri*. ^1^H NMR spectroscopy was used to quantify reactants and products before and after a 3-day fermentation period. The results (Figures 4a and S2) showed a clear depletion of acetate and ethanol concentrations and an increase in butyrate and caproate concentrations within the media. This confirmed that *C. kluyveri* was effectively capable of the desired conversion, producing C_4_ and C_6_ chemicals from C_2_ feedstocks. To validate this concept using the solar-derived products, the post-BPEC media was taken directly after 140 h to initiate fermentation. After the BPEC reaction, the media contained 228 ± 24.6 μM of ethanol and 224.8 ± 18.3 μM of acetate. *S. ovata* cells were removed by filtration (0.2 μm), and 10 mL of the filtered supernatant was used per fermentation vial. The vials were sealed and purged with 80% N_2_: 20% CO_2_ (pH 7.0). *C. kluyveri* were cultured using DSMZ 556 media (Table S5) before adding *C. kluyveri* cells (without any additional ethanol or acetate) to an OD_600_ = 0.5–0.7. Fermentation was proceeded for 3 days at 37 °C. ^1^H NMR confirmed the initial presence of “solar-acetate” and “solar-ethanol,” with no detectable butyrate or caproate at time zero. After 3 days of fermentation, ^1^H NMR revealed that butyrate (C_4_) and caproate (C_6_) were produced while acetate and ethanol concentrations were depleted (Figure 4c). The butyrate and caproate concentrations increased as expected via fermentation, with 1.31 ± 0.2 μmols of butyrate and 0.6 ± 0.1 μmols of caproate, and 0.72 ± 0.2 μmols of H_2_ were produced (Figure 4b,c). The conversion efficiencies, based on ethanol consumption and stoichiometry from eq 1, were calculated to be 49% for C_4_, 62% for C_6_, and 60% for H_2_. This indicates that 49% of the theoretical maximum amount of butyrate was produced relative to the consumed acetate and ethanol. Similarly, 62% of caproate and 60% of H_2_ are calculated independently using the same stoichiometric basis. These values suggest a kinetic bottleneck within the chain elongation metabolism, preventing a total conversion of precursors. Furthermore, it is likely that some ethanol and acetate are used for sustaining life via alternative metabolic pathways.

## Conclusions

In this study, we present a semiartificial biohybrid platform that enables the efficient conversion of CO_2_ into valuable MCFAs using an earth-abundant and scalable BPEC system. A conversion that remains unattainable using an abiotic system has been made possible through the catalytic precision of microbial metabolism. The system leverages the advantages of both inorganic semiconductors and the selectivity of naturally evolved organisms developed over billions of years of evolution. *S. ovata* was interfaced with a CuBi_2_O_4_|MgO photocathode, which exhibits excellent visible-light absorption and stability over an extended operational period of more than 140 h. *S. ovata* was chosen due to its CO_2_-fixing and electrotrophic characteristics, as well as its ability to produce ethanol and acetate as metabolites from CO_2_. The CuBi_2_O_4_ photocathode was passivated with an MgO layer to produce reducing equivalents, which are taken up by *S. ovata* for CO_2_ reduction without the need for SEDs. This system marks a significant improvement over current state-of-the-art microbial colloidal photocatalytic systems, which contain expensive SEDs that complicate mechanistic interpretation and introduce inefficiencies. The metabolism from live *S. ovata* cells converted CO_2_ directly into two known metabolites, acetate and ethanol, using photogenerated electrons as the electron donors. Similar quantities of photosynthesized acetate and ethanol were observed after the 140 h BPEC reaction. Here, the traditional barriers in CO_2_RR catalysis posed by the multiple PCET transfer steps, which make producing multicarbon products in an abiotic system incredibly challenging, have been overcome. The solution processability of the CuBi_2_O_4_|MgO makes the photocathodes amenable to scale. These methods could be extended to larger electrode areas with modest fabrication costs compared to high-vacuum deposition techniques. Regarding microbial stability, *S. ovata* cultures in our study remained viable and active for over 140 h, consistent with prior reports of their long-term robustness under bio-PEC conditions. Nonetheless, at larger scales, sustained microbial viability will likely require optimization of bioreactor design, nutrient delivery, product extraction, and purification. Finally, O_2_ accumulation is a critical challenge for scaling up hybrid bio-PEC systems. While significant O_2_ accumulation was not observed in our lab-scale reactions, in larger, more efficient upscaled systems, O_2_ levels above 10% will need additional solutions to prevent the inhibition of the anaerobic microbes. Engineering solutions such as gas-permeable membranes and compartmentalized bioreactors are potential solutions. The C_2_ metabolites were subsequently fermented by *C. kluyveri* into butyrate, caproate, and H_2_. The combination of these two biochemical transformations demonstrates a novel and clean route for the conversion of waste CO_2_ into industrially relevant chemicals, with huge implications for the sustainable production of biofuels and surfactants, moving away from current carbon-heavy methods of chemical production.

## Materials and Methods

### Culturing of *S. ovata*

*S. ovata* was purchased directly from the Leibniz Institute DSMZ–German Collection of Microorganisms and Cell Cultures GmbH (DSMZ no. 2662). *S. ovata* were cultured using CO_2_ as the electron acceptor and betaine as the electron donor. The culture medium was the recommended growth medium (DSMZ311), omitting casitone, Na-resazurin, and Na_2_S (Table S1). To keep anaerobic conditions, the medium was purged with a gas mixture of 80% N_2_ and 20% CO_2_ and purged into the vials for 45 min. Inoculated cultures were then incubated at 30 °C at 200 r.p.m. Cultures were grown to an OD_600_ = 0.5–0.7 and monitored using ^1^H NMR (Bruker 400 MHz) to track acetate production with 3-(trimethylsilyl)­propionic-2,2,3,3-d4 acid sodium salt (TSP) as an internal standard in D_2_O (Figure S1).

### Culturing of *C. kluyveri*

*C. kluyveri* was purchased from the Leibniz Institute DSMZ–German Collection of Microorganisms and Cell Cultures GmbH (DSMZ no. 556). *C. kluyveri* was cultured using acetate and ethanol as a feedstock for fermentation. The culture medium was an adapted recommended DSMZ 52 medium (Table S2). Anaerobic conditions were maintained using a gas mixture composed of 80% N_2_ and 20% CO_2_, which was purged into the vials for 45 min. Cultures were grown at 37 °C to an OD_600_ = 0.5–0.7 and monitored using ^1^H NMR (Bruker 400 MHz) to track acetate/ethanol consumption and butyrate (C_4_) and caproate (C_6_) production with TSP in D_2_O as an internal standard (Figure S2).

### Photocathode Fabrication

CuBi_2_O_4_ nanoparticles were synthesized as per our previous work.^31^ Briefly, CuBi_2_O_4_ nanoparticles were synthesized in an automated coprecipitation reactor. Cu^2+^ and Bi^3+^ precursors were pumped in a controlled manner to allow stoichiometric control (2:3, respectively) of the metal hydroxide nanoparticles. NaOH, citric acid, and deionized water were also fed in using HPLC pumps, controlling the flow rate to maintain a pH of 12. The high concentration of OH^–^ ions leads to hydrolysis and dehydration of the Cu and Bi salts, precipitating a green solid, CuBi_2_(OH)_4_, which was then centrifuged and washed with DI water 3×. The product was then dried overnight in a vacuum desiccator. The dried product was sintered in air at 650 °C for 1 h to complete oxidation to CuBi_2_O_4_. The nanoparticles were ground in a Retsch PM100 planetary ball mill for 12 h in ethanol and filtered through 20 mm gauze to decrease particle size and increase the uniformity of particle size. A paste was then fabricated using a mixture of terpineol (5g), and ethyl cellulose (6g) along with 2 g of the CuBi_2_O_4_. Excess ethanol was then evaporated under reduced pressure until the desired paste thickness was achieved. Films were deposited onto clean FTO glass via blade-coating and annealed at 450 °C. MgO was deposited on the surface from a solution of 1:50 Mg­(CH_3_COO^–^)_2_·*X*H_2_O via spin coating 200 mL onto a 2 cm × 2 cm film at 2000 rpm for 30 s and annealing at 450 °C for 30 min.

### Photoelectrochemical Reactions

PEC/BPEC reactions were carried out in closed 3-neck flasks with illumination through the side of the vessel. Illumination was performed using Newport Oriel 67005 solar simulators (300W Xe, 100 mW cm^–2^, AM1.5G filtered). The lamp was calibrated using a Newport 1916-R optical power meter. Photocathode samples (0.25 cm^2^) were suspended in electrolyte using copper tape, combined, and attached to a Ti rod to enable connection to a Potentiostat. The electrolyte for PEC/BPEC reactions (25 mL) contained (DSMZ311) *S. ovata* medium excluding Na_2_S, casitone, betaine, l-cysteine, yeast, and Na-resazurin (unless otherwise mentioned). Prior to performing any CO_2_RR, the cell was purged with CO_2_:N_2_ (20%:80%) for 45 min to provide a CO_2_-rich anaerobic environment. *S. ovata* cells were then added via injection to a final pH 6.8–7.2 and an OD_600_ of 0.5–0.7. The PEC setup is shown in Figure S3.

### Fermentation with *C. Kluyveri*

Chain elongation of acetate and ethanol (produced by *S. ovata* *) was carried out using* *C. kluyveri*, which has the capacity to produce butyrate and caproate. After the BPEC reaction with the biohybrid system, the media was filtered through a 20 mm filter to remove *S. ovata* cells. The media (5 mL) was then sealed and purged with CO_2_/N_2_ (20%:80%). *C. kluyveri* cells (which had been previously cultured with ethanol and acetate as carbon sources) were added (OD_600_ = 0.3–0.5) and monitored using ^1^H NMR (Bruker 400 MHz) to track acetate/ethanol consumption and butyrate (C_4_) and caproate (C_6_) production with TSP in D_2_O as an internal standard (Figure S4).

### Product Quantification

Concentrations of liquid carbon products were measured using ^1^H NMR spectroscopy on a 400 MHz Bruker Spectrometer. In each sample, 0.2 mL of 10 mM TSP in D_2_O was added to 0.8 mL of the filtered sample. Spectra were analyzed using Mestrenova Software. H_2_ evolution was quantified using a Shimadzu gas chromatograph equipped with a thermal conductivity detector at 250 °C. Representative GC traces of a standard gas mixture (Figure S5).

### Structural, Morphological, and Optical Characterization

Film morphology was studied using a Tescan Mira3 scanning electron microscope (SEM). Energy-dispersive X-ray analysis (EDS) was carried out using the SEM and was analyzed using an Oxford Instruments EDS analyzer. Film structure was determined by X-ray diffraction (XRD) of the thin films on FTO glass using a Rigaku SmartLab X-ray diffractometer. The absorption spectra of films were recorded using an Ocean Optics fiber optic setup connected to an LS-1 light source and a USB2000 detector.

### BET Analysis

BET adsorption/desorption isotherms were acquired on films of CuBi_2_O_4_ with a Micromeritics ASAP 2420 instrument, using Krypton as the adsorbate. The CuBi_2_O_4_ films on FTO glass substrates were weighed and placed into a sample tube and degassed at 150 °C for 15 h under high vacuum (<0.013 mbar) to remove moisture and other adsorbed gases. The Krypton isotherms (at −197 °C, 0.12–0.65 relative pressure) generated were the result of the average of 3 separate samples. Average isotherms associated solely with the CuBi_2_O_4_ films were calculated by subtraction of blank isotherms on an empty sample tube and the FTO glass substrate, and the weight was adjusted to remove the FTO glass. Specific surface area, micropore volume, and limited mesopore volume (from 2–20 nm) were calculated using the BET model (eq S1) and Derjaguin–Broekhoff–de Boer model.^46^

Within the BET eq (eq S3), *P* = partial pressure of the adsorbate gas; *P*_0_ = saturation pressure of the adsorbate gas; *V* = volume of adsorbed gas; *V*_m_ = monolayer adsorbed gas volume; *C* = BET constant (the C parameter).

### Electrochemical Surface Area

The electrochemical surface area (ECSA) of the CuBi_2_O_4_|MgO electrodes was determined using cyclic voltammetry (CV) in a non-Faradaic region. Measurements were conducted in a three-electrode electrochemical cell using CuBi_2_O_4_|MgO as the working electrode, a platinum wire as the counter electrode, and an Ag/AgCl (3.5 M KCl) reference electrode. The electrolyte solution consisted of *S. ovata* medium (pH 7). Cyclic voltammograms were recorded at scan rates ranging from 10 to 500 mV s^–1^. The electrochemical was determined by plotting the difference in anodic and cathodic current densities (Δ*j* = *j*_anodic – *j*_cathodic) at a fixed potential against the scan rate. The slope of the linear fit was used to calculate the double-layer capacitance (*C*_DL_), which was then correlated with ECSA using eqs S2 and S3.

Specific capacitance (*C*_e_) is typically assumed to be 20–60 mF cm^–2^ for metal oxides. The measurements were performed in triplicate for each electrode to ensure reproducibility.

### Electrochemical Impedance Spectroscopy

Electrochemical impedance spectroscopy (EIS) was performed using a three-electrode configuration in a potentiostat (Biologic, VMP3e) with CuBi_2_O_4_|MgO as the working electrode, a platinum wire as the counter electrode, and an Ag/AgCl (3.5 M KCl) reference electrode. The measurements were conducted in an aqueous electrolyte solution (*S. ovata* medium, pH 7) under both dark and illuminated conditions using a calibrated solar simulator (Xe lamp, AM 1.5G, 100 mW cm^–2^). Nyquist and Bode plots were recorded by applying an AC perturbation of 10 mV amplitude over a frequency range of 0.01 Hz to 200 kHz at different DC biases (0 and −0.2 V vs Ag/AgCl). All measurements were repeated in triplicate to ensure reproducibility.

### Raman Spectroscopy

Raman spectroscopy measurements were conducted using the Edinburgh Instruments Raman Microscope to analyze the vibrational modes of various samples. The system utilizes a confocal Raman microscope equipped with a high-sensitivity CCD detector and a choice of laser excitation sources, which allows for detailed molecular characterization with high spatial resolution. Raman measurements were performed using a 532 nm laser source for excitation. The system was configured with a 20× objective lens, which was employed to achieve high spatial resolution and maintain a strong signal-to-noise ratio. The spectrometer acquired data from 0 cm^–1^ to 4000 cm^–1^. Spectra were acquired with an exposure time of 1s and 15 accumulations. The laser power was adjusted to minimize sample damage, typically operating at 5–10 mW (10–20%) depending on the sample’s sensitivity to laser irradiation, aiming to enhance the signal-to-noise ratio.

### Confocal Laser Scanning Microscopy (CLSM) LIVE-DEAD Assay

CuBi_2_O_4_|MgO|*S. ovata* composites, prepared via biophotoelectrochemical (BPEC) reaction under the same controlled conditions as mentioned in “Photoelectrochemical Reactions” methodology. Following the BPEC reactions, the samples were rinsed with phosphate-buffered saline (PBS) to remove residual solution and suspended impurities, ensuring that only the cells of interest remained on the surface. The samples were then placed in a Petri dish, ready for imaging. A LIVE/DEAD BacLight Bacterial Viability Kit (Invitrogen) was utilized to confirm the viability of *S. ovata* cells after BPEC treatment for over 5 days. This assay functions on a selective staining basis, with two different fluorescent dyes: (SYTO 9 and propidium iodide (PI)). SYTO 9 is a green-fluorescent dye that stains viable cells, while PI stains only those cells with compromised membranes, resulting in red fluorescence for dead cells.

## Supplementary Material



## Notes

The data set includes product quantification, SEM images, BET pore size distributions, electrochemical surface area, electrochemical impedance spectroscopy, XRD, CLSM LIVE/DEAD assays, XPS, Raman spectroscopy, post-PEC characterization, media composition, and comparison tables. Original data is available upon reasonable request (PDF)
