# Supplementary material for: A Living Semiartificial Photoelectrocatalytic Biohybrid for Solar CO2 Fixation and Fermentation to Fatty Acids
Source: ACS Appl Mater Interfaces. 2025 Nov 10;17(46):63363–73. doi: 10.1021/acsami.5c15023 (PMC12635957; doi:10.1021/acsami.5c15023)
Supplement: Supplementary file 1 [file am5c15023_si_001.pdf]

# Supporting Information

## A Living Semi-Artificial Photoelectrocatalytic Biohybrid for Solar $CO_2$ Fixation and Fermentation to Fatty Acids

Cathal Burns[1,2], Muhammed Rishan[1,2], Lee Stevens[3], Ellie Ashcroft[1], Linsey Fuller[4], Elizabeth A Gibson[2], and Shafeer Kalathil[1]*

*Corresponding Author

Email: shafeer.kalathil@northumbria.ac.uk

1. *Faculty of Science and Environment, School of Geography and Natural Sciences, Northumbria University, Newcastle, NE1 8ST, United Kingdom*

2. *School of Natural and Environmental Sciences, Newcastle University, Newcastle upon Tyne, NE1 7RU, United Kingdom*

3. *Low Carbon Energy Research and Technologies Group, Faculty of Engineering, University of Nottingham, Nottingham, NG7 2TU, United Kingdom*

4. *Procter and Gamble Innovation Centre, Whitley Rd, Newcastle upon Tyne, NE12 9TS, United Kingdom*

**Supporting Figures**

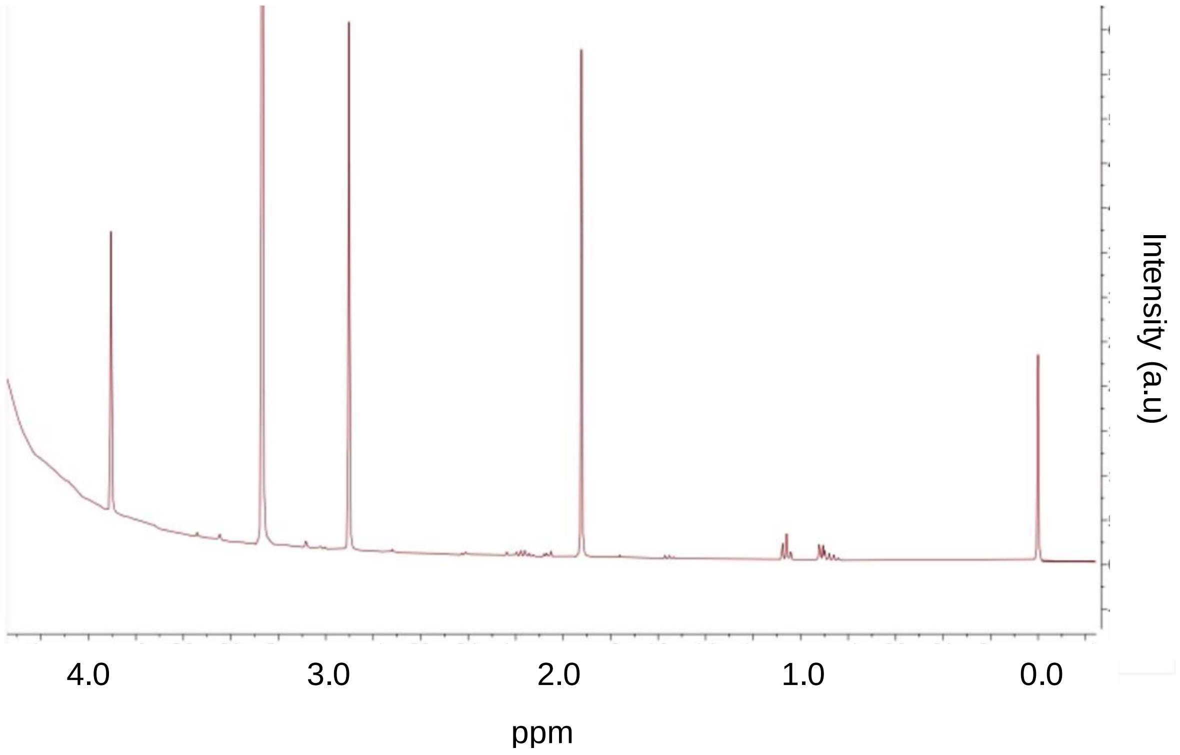


**Figure S1**– A typical $^{1}$H NMR after 3-days of growth of *S. ovata* in betaine rich media. Acetate is the major product (s, 1.9 ppm), with small traces of ethanol present (t, 1.1 ppm)

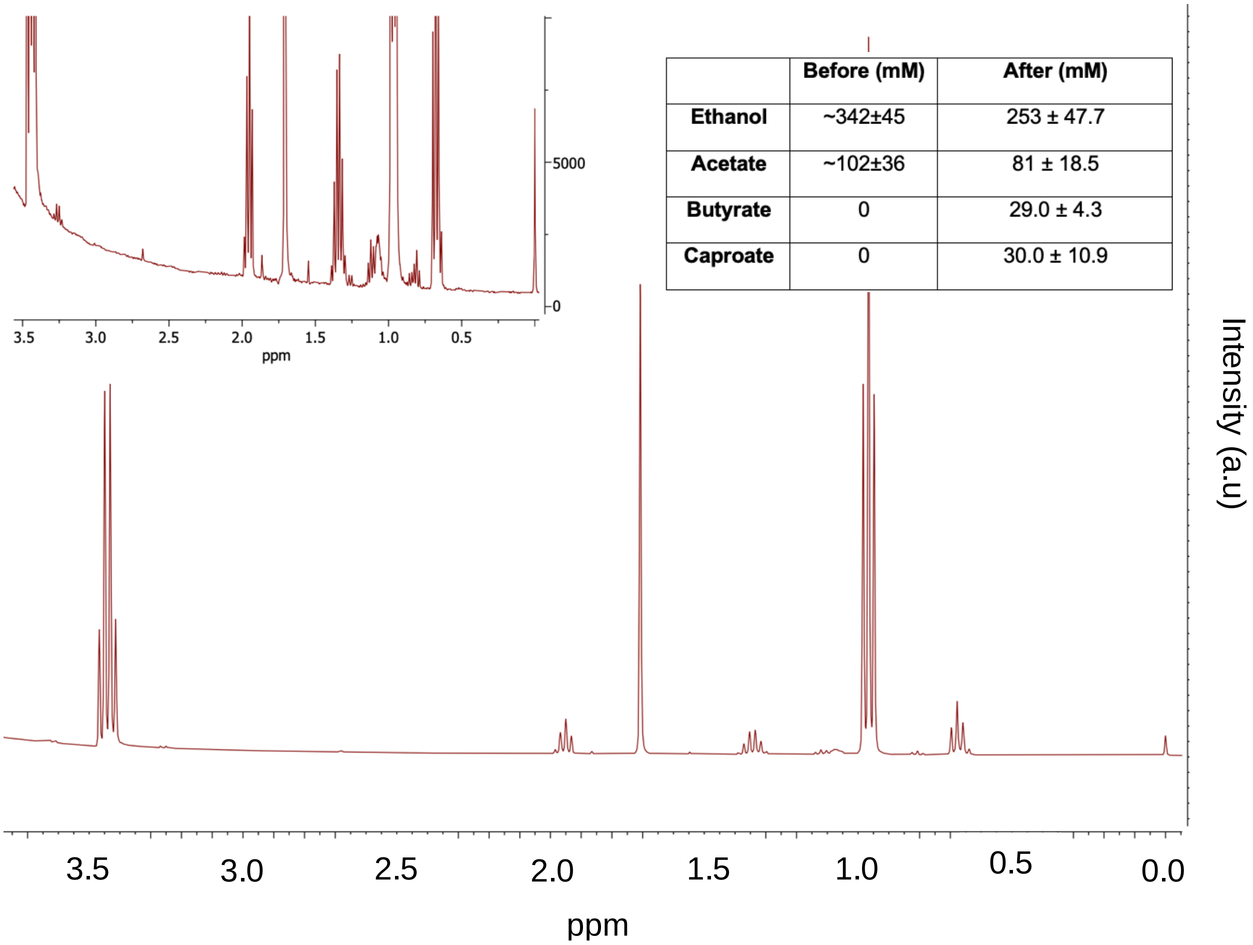


| | Before (mM) | After (mM) |
|---|---|---|
| **Ethanol** | ~342±45 | 253 ± 47.7 |
| **Acetate** | ~102±36 | 81 ± 18.5 |
| **Butyrate** | 0 | 29.0 ± 4.3 |
| **Caproate** | 0 | 30.0 ± 10.9 |

**Figure S2** – A typical $^1$H NMR of *C. kluyveri* after culturing in $N_2$:$CO_2$ (80:20). Inset: A summary of concentrations detected before and after a 3-day culturing of *C. kluyveri* with ethanol and acetate. $^1$H NMR (400 MHz, $D_2O$) **Ethanol**: δ 0.95 (t, 3H), 3.45 (q, 2H). **Acetate:** δ 1.70 (s, 3H). **Butyrate:** δ 0.65 (t, 3H), 1.35 (m, 2H), 1.95 (t, 2H). **Caproate:** δ 0.76 (t, 3H), 1.10 (m, 4H), 1.35 (m, 2H), 1.95 (t, 2H). Inset: zoomed $^1$H NMR spectrum between δ 0 – 3.6.

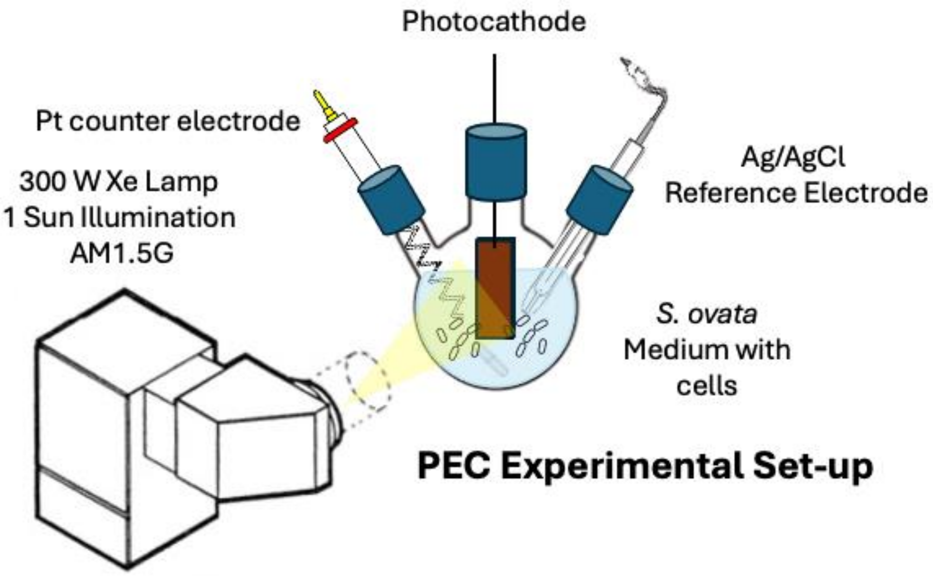


**Figure S3.** Experimental set-up of a typical PEC reaction with photocathode as working electrode, Pt wire as a counter electrode, Ag/AgCl as reference electrode, *S. ovata* medium as the electrolyte, $OD_{600}$ = 0.5-0.7 of *S. ovata* cells, a 300W Xe lamp calibrated to 1 sun illumination (AM1.5G), the atmosphere inside the sealed reactor was 80%:20% $N_2$:$CO_2$ and a magnetic stirrer bar set to 300 rpm.

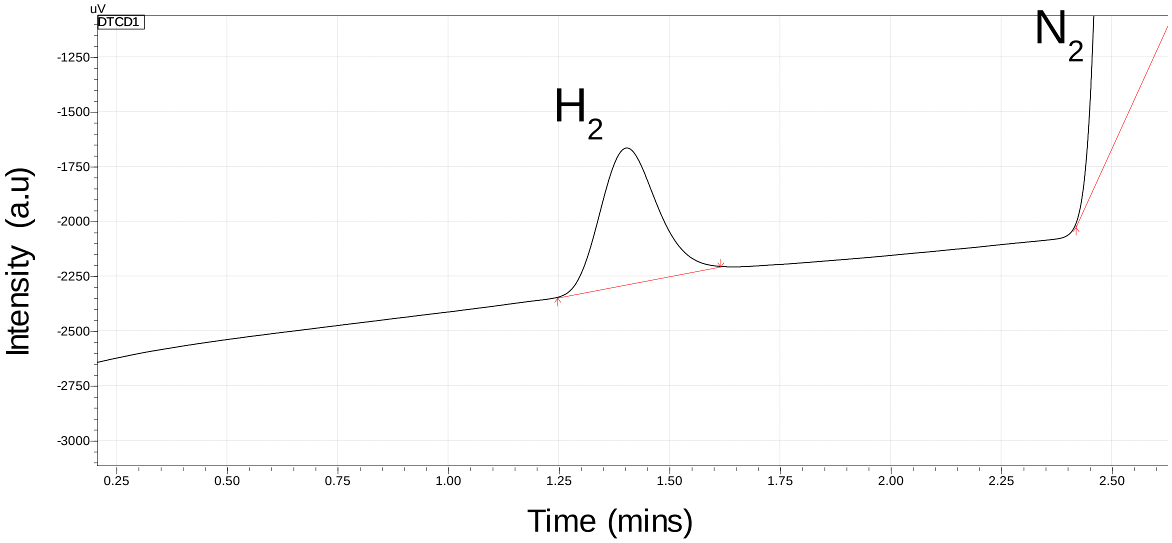


**Figure S4**. Representative GC trace of typical headspace analysis after a 3-day fermentation reaction of the photosynthesized acetate and ethanol with *C. kluyveri*.

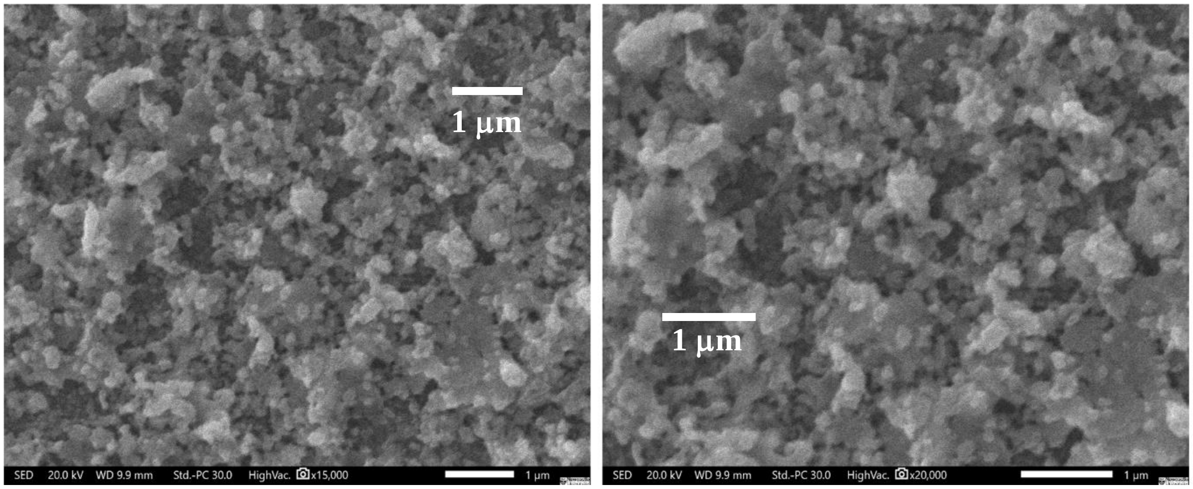


**Figure S5.** SEM images of $CuBi_2O_4$ films on FTO glass substrates before any (photo)electrochemical reaction. Left: Magnification 15 kx, and working distance (WD) = 9.9 mm. Right: Magnification 20 kx, and working distance (WD) = 9.9 mm. A secondary electron detector was used.

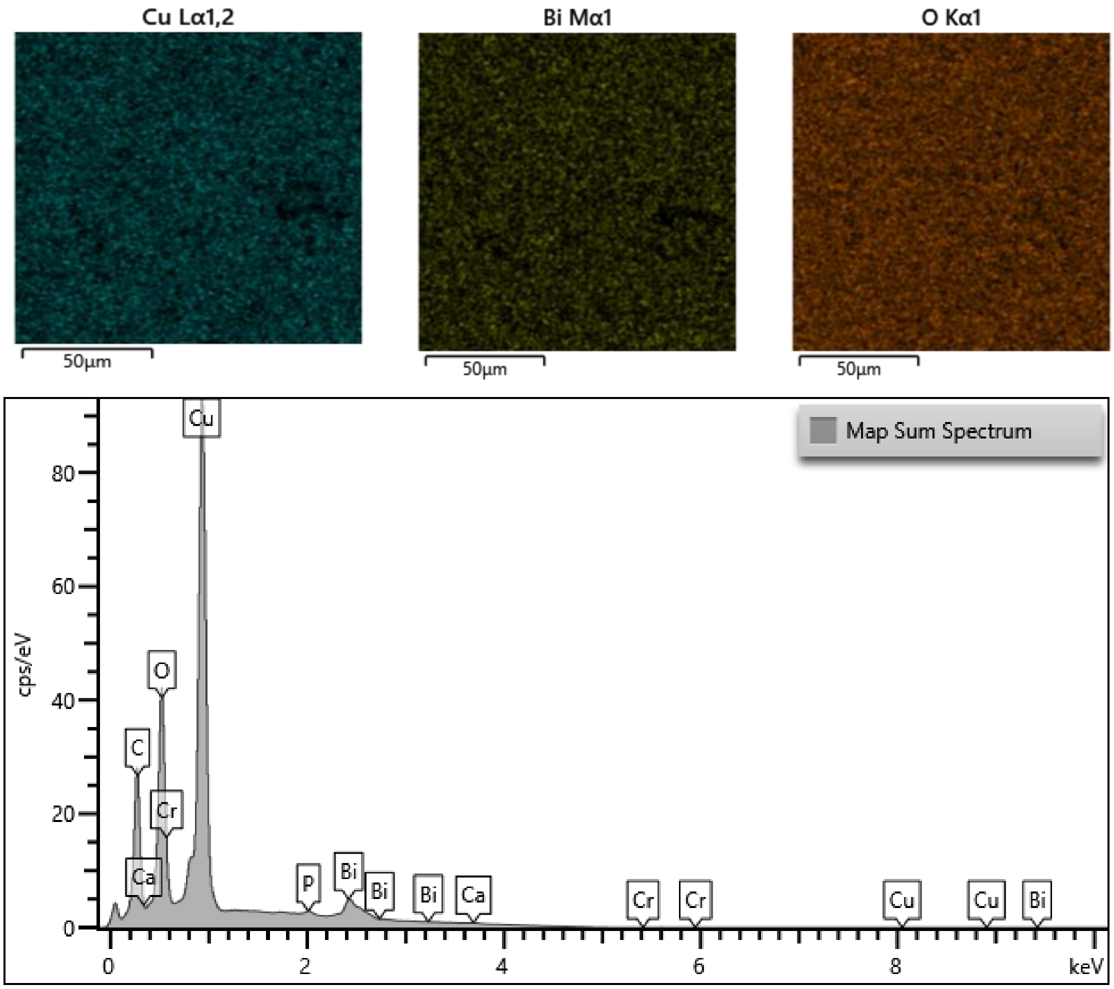


**Figure S6 –** SEM-EDS of the $CuBi_2O_4$|MgO|*S.ovata* photocathode post 140-hour PEC reaction showing the presence of Cu, Bi, O, C, Ca, and P.

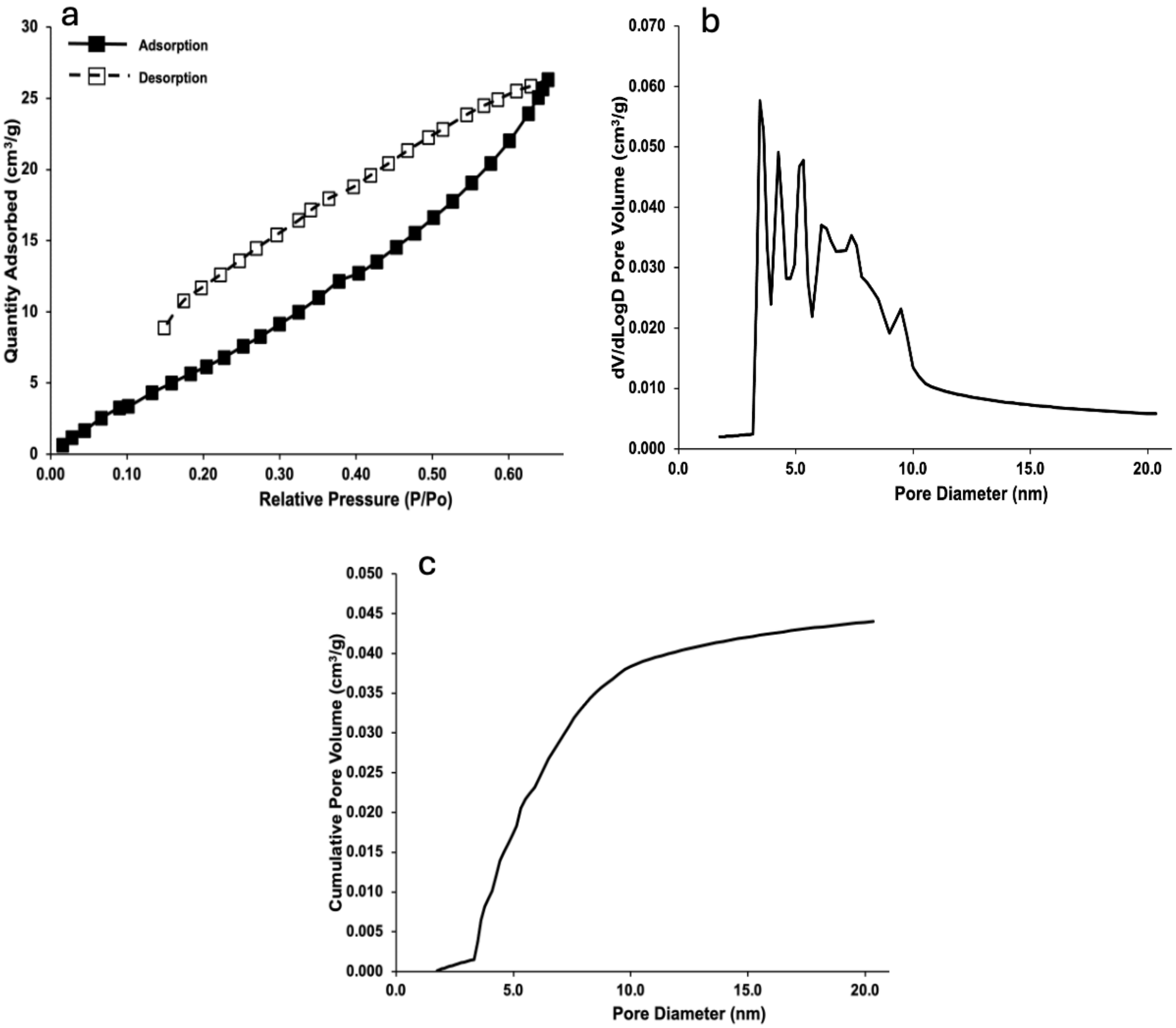


**Figure S7 – Krypton adsorption-desorption isotherm and pore size distribution analysis of $CuBi_2O_4$. -** ***(a)*** Krypton adsorption-desorption isotherm, showing the quantity of Krypton adsorbed (black squares) and desorbed (open squares) as a function of relative pressure ($P/P_0$). The isotherm (Type IVa) suggests a mesoporous material with a characteristic hysteresis loop, indicative of capillary condensation. ***(b)*** Differential pore size distribution derived from the Derjaguin–Broekhoff–de Boer model. ***(c)*** Cumulative pore volume as a function of pore diameter.

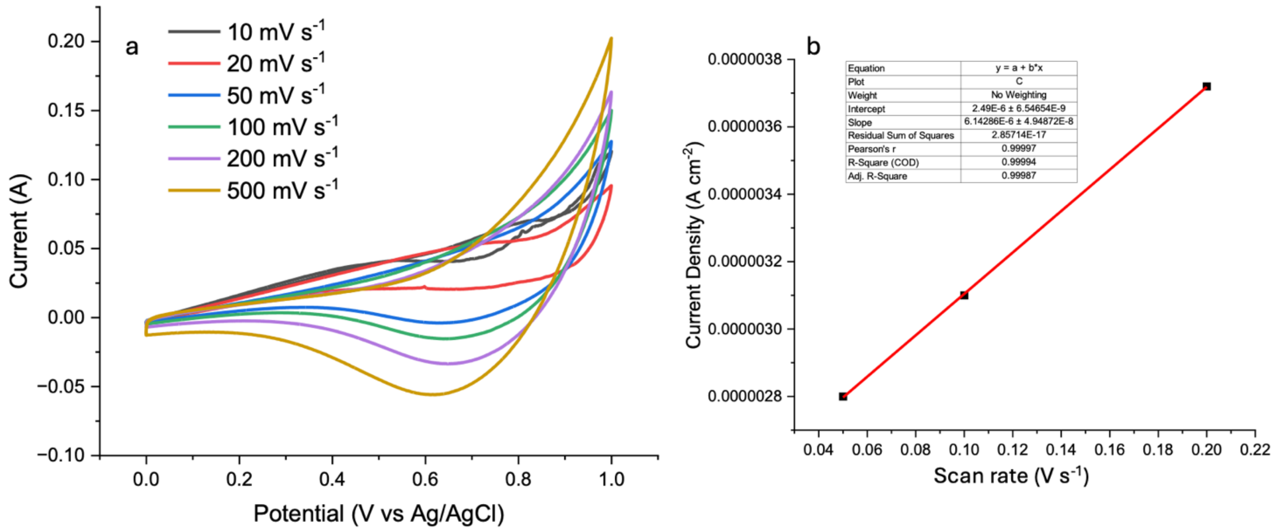


**Figure S8 – Electrochemical Surface Area *(a)*** Cyclic voltammograms recorded at different scan rates (10, 20, 50, 100, 200, and 500 mV $s^{-1}$) using a working electrode of $CuBi_2O_4$|MgO on an FTO substrate in an electrochemical system with Pt wire as a counter electrode and a 3.5M Ag/AgCl reference electrode. The current (A) is plotted against the potential (V vs Ag/AgCl). ***(b)*** Corresponding plot of current density (A $cm^{-2}$) vs. scan rate (u) with a linear fit, the slope of which is equal to the capacitance of the double layer ($C_{DL}$). The inset table provides the equation of the fitted line, $R^2$ value, and other relevant statistical parameters.

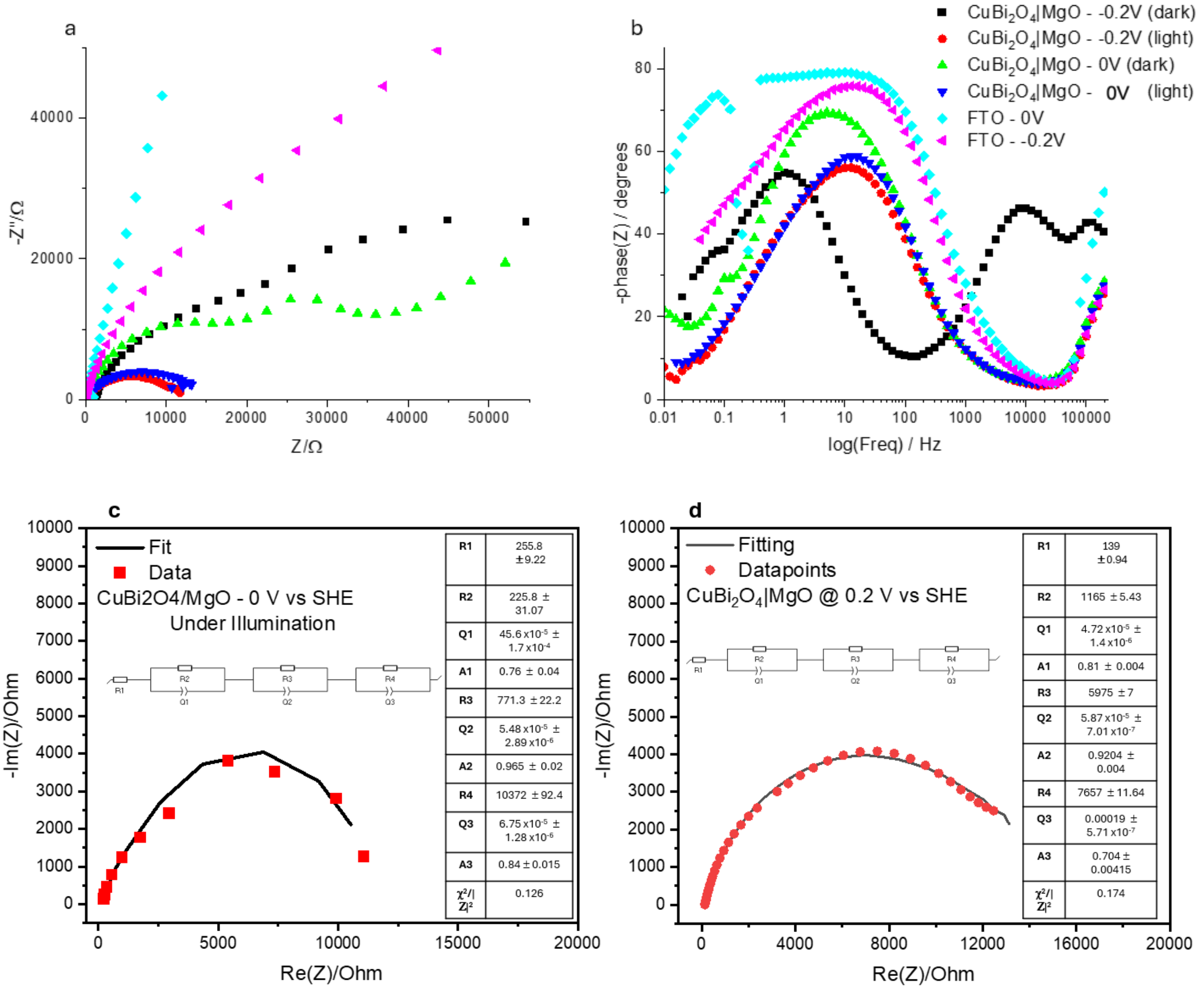


**Figure S9 – Electrochemical impedance spectroscopy (EIS) analysis of $CuBi_2O_4$|MgO electrodes under different conditions.** - ***(a)*** Nyquist plots displaying the real (Z) and imaginary (Z'') components of impedance for $CuBi_2O_4$|MgO at applied biases of -0.2 V and 0 V vs Ag/AgCl, under both dark and illuminated conditions (1 sun, AM1.5G, 100 mW $cm^{-2}$), as well as for the FTO substrate as a control. ***(b)*** Bode phase plots showing the phase angle as a function of frequency. ***(c–d)*** Nyquist plots of electrochemical impedance spectroscopy (EIS) measurements of $CuBi_2O_4$/MgO photocathodes under illumination. **(c)** At 0 V vs SHE showing experimental data (red squares) and fitting (black line), along with the equivalent circuit model and fitted

parameters. **(d)** At 0.2 V vs SHE with experimental data (red circles) and fitting (black line), also including the corresponding equivalent circuit and fitted parameters.

The equivalent circuit used to fit the electrochemical impedance spectroscopy (EIS) data consisted of the solution / uncompensated resistance (R1), a high frequency arc ($R_2/Q_1$) is dominated by the bulk of the $CuBi_2O_4$ and the MgO / FTO interfaces. The mid frequency arc ($R_3/Q_2$) represents charge transfer between $CuBi_2O_4$ and the electrolyte, strongly influenced by surface states at the $CuBi_2O_4$|MgO/ electrolyte interface. The low frequency arc ($R_3/Q_2$) represents transport-limited / slow surface processes. This captures the slowest ste, the Nerst diffusion of reactants/products near the electrode and /or very slow surface-state/ trap dynamics.

Electrochemical impedance spectroscopy (EIS) was employed to investigate the interfacial charge transfer dynamics and electrochemical behaviours of the $CuBi_2O_4$|MgO films within *S. ovata* medium as the electrolyte ( **Figure 5a-b**). Experiments were carried out under illumination and in the dark as well as at varied applied potentials (0.2V and 0V vs SHE). At 0.2V vs SHE, the dark experiments revealed a relatively high charge transfer resistance ($R_{ct}$) as expected, due to the limited availability of free charge carriers. The Nyquist plot consists of a large semicircle which is indicative of high interfacial resistance, limiting charge transfer. The Bode phase plot shows a peak at ~ 10 Hz which suggests dominant capacitive behaviour at the interface. Upon illumination, the EIS spectra change significantly as the absorption of photons of sufficient energy to bridge the bandgap generates electron-hole pairs. This leads to a reduction in the $R_{ct}$ which is indicated in the contracted smaller semicircle in the Nyquist plot, suggesting enhanced charge transfer

efficiency. The Bode plot also displays a slight shift to higher frequencies which reflects the reduction in recombination and faster carrier dynamics. At 0V vs SHE under dark conditions, a similar effect was observed. However, upon the application of a slight cathodic bias, the system is biased towards cathodic charge accumulation leading to an increase in $R_{ct}$ due to charge injection being hindered by the bulk recombination within the $CuBi_2O_4$|MgO architecture. This is observed in the Nyquist plot with a larger semicircle observed than at 0.2V vs SHE. Upon illumination, photogenerated excitons significantly decrease $R_{ct}$ with the Nyquist plot showing a pronounced decrease in semicircle size.

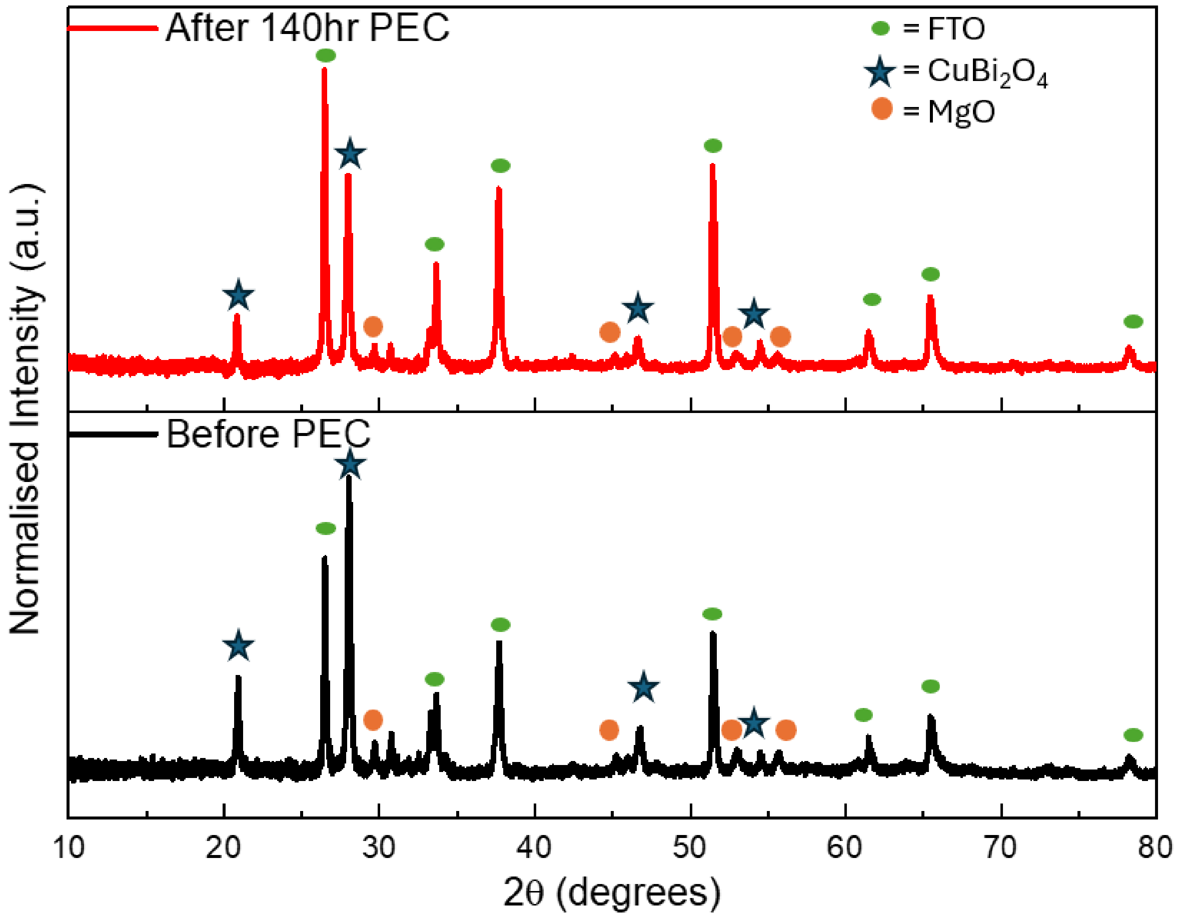


**Figure S10** – XRD diffractograms of $CuBi_2O_4$|MgO films on FTO glass substrates before and after the 140-hour PEC reaction. FTO is indicated by the green circles, MgO by the orange circles, and $CuBi_2O_4$ by the blue stars

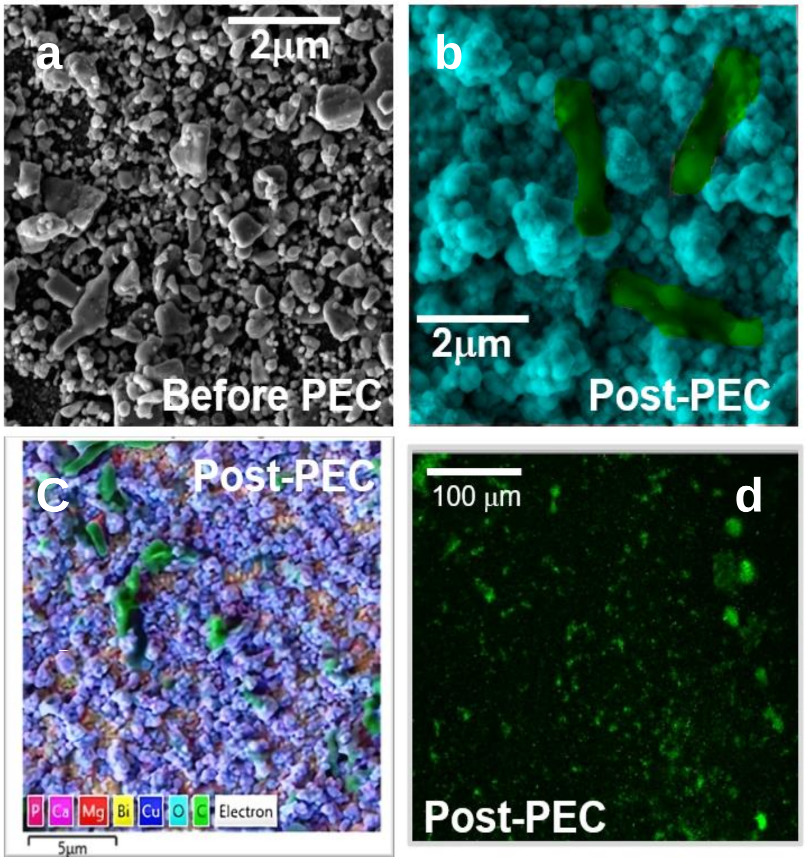


**Figure S11. Biohybrid Morphology. a)** Top-view SEM of $CuBi_2O_4$|MgO before BPEC. View field = 6.86 μm, magnification 34.2 kx, and working distance (WD) = 12.7 mm. **b)** Top-view SEM of $CuBi_2O_4$|MgO|*S. ovata* after 140 hours of BPEC. View field = 6.85 μm, magnification 34.3 kx, and working distance (WD) = 7.53 mm. *S. ovata* cells highlighted in green and $CuBi_2O_4$|MgO in blue. **c)** Top view SEM-EDX showing an elemental map of the CuBi2O4|MgO|*S. ovata* biohybrid after 140 hours of BPEC. Green corresponds to carbon and shows the presence of *S. ovata* cells at the inorganic interface. View field = 20.6 μm, magnification 5.7 kx, and working distance (WD) = 7.53 mm. A secondary electron detector was used for all images. **d)** CLSM image of a LIVE/DEAD assay on $CuBi_2O_4$|MgO|*S. ovata* photocathode after 140 hours of BPEC. The biohybrid

photocathodes were stained with SYTO 9 (2 μM) and propidium iodide (PI, 2 μM) and incubated in the dark for 30 min at 20 °C. Excitation: $\lambda_{ex}$ = 488 nm, emission: $\lambda_{em}$ = 530-543 for SYTO 9 and 610-620 nm for PI.

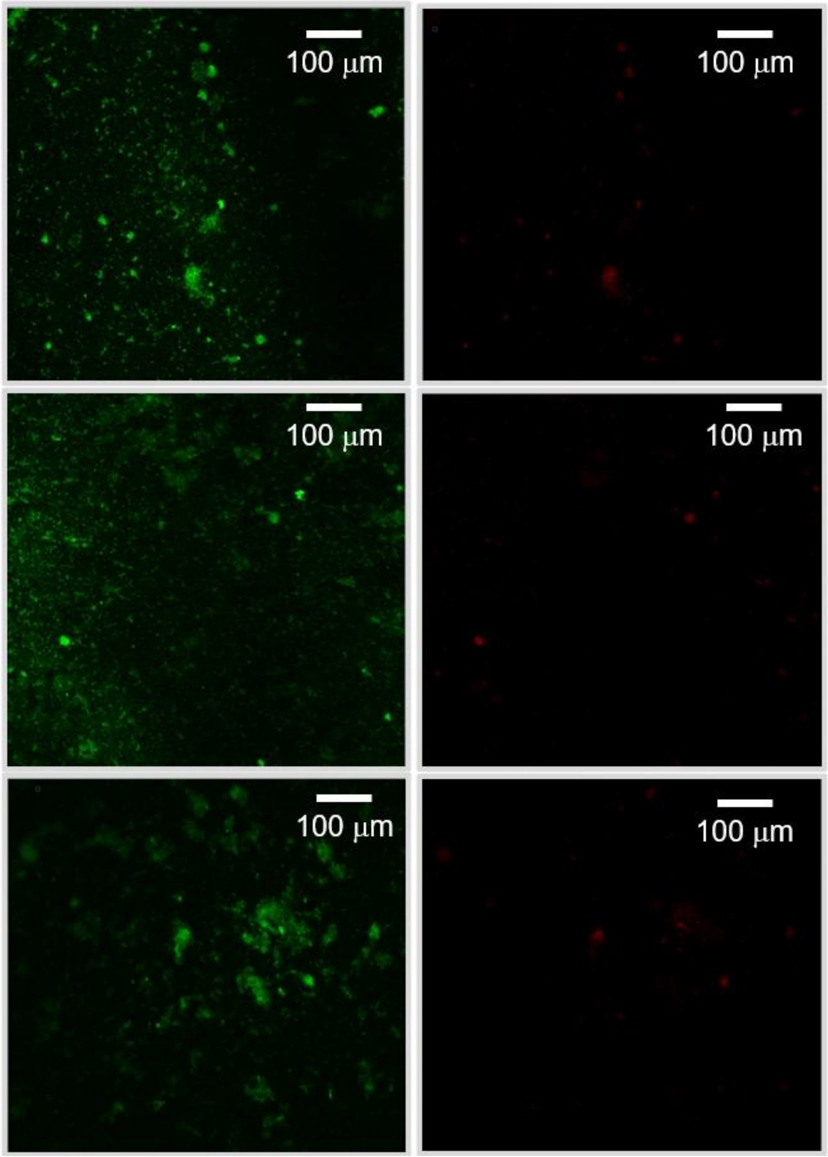


**Figure S12** – CLSM images of a LIVE/DEAD assay on $CuBi_2O_4$|MgO|*S. ovata* photocathodes after 140 hours of PEC. The biohybrid photocathodes were stained with SYTO 9 (2 mM) and propidium iodide (PI, 2 mM) and incubated in the dark for 30 min at 20 °C. Excitation: $\lambda_{ex}$ = 488 nm, emission: $\lambda_{em}$ = 530-543 for SYTO 9 and 610-620 nm for

PI. Red fluorescence indicates dead cells, and green fluorescence indicates viable (alive) cells.

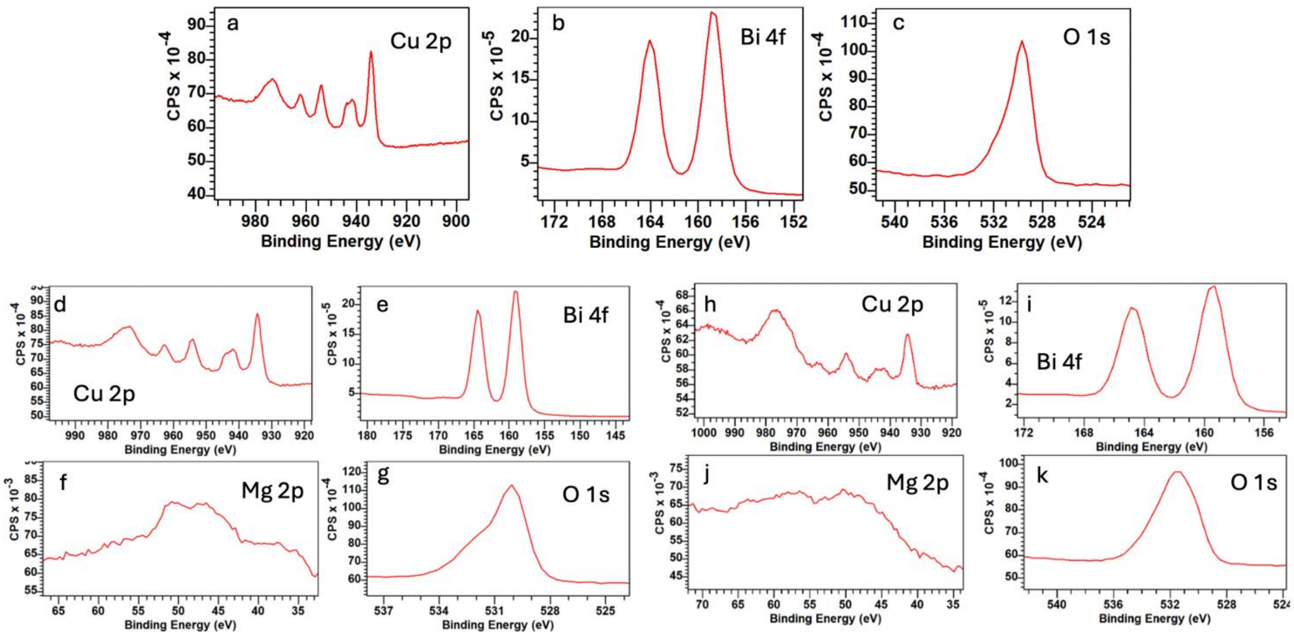


**Figure S13 –** X-ray photoelectron spectroscopy (XPS) analysis of $CuBi_2O_4$-based photocathodes. **(a–c)** XPS spectra of $CuBi_2O_4$, showing the Cu 2p, Bi 4f, and O 1s core levels, consistent with $Cu^{2+}$, $Bi^{3+}$, and lattice oxygen states. **(d–g)** XPS spectra of $CuBi_2O_4$|MgO prior to photoelectrochemical (PEC) operation, highlighting the preserved Cu and Bi oxidation states, the presence of Mg 2p contributions, and a broadened O 1s peak due to mixed oxygen environments. **(h–k)** XPS spectra of $CuBi_2O_4$|MgO after PEC operation, revealing attenuation of Cu 2p intensity (suggestive of partial Cu reduction), stable but diminished Bi 4f features, persistence of Mg-related signals, and a shift/broadening of the O 1s peak toward higher binding energies, indicative of surface hydroxylation and defect formation during PEC testing.

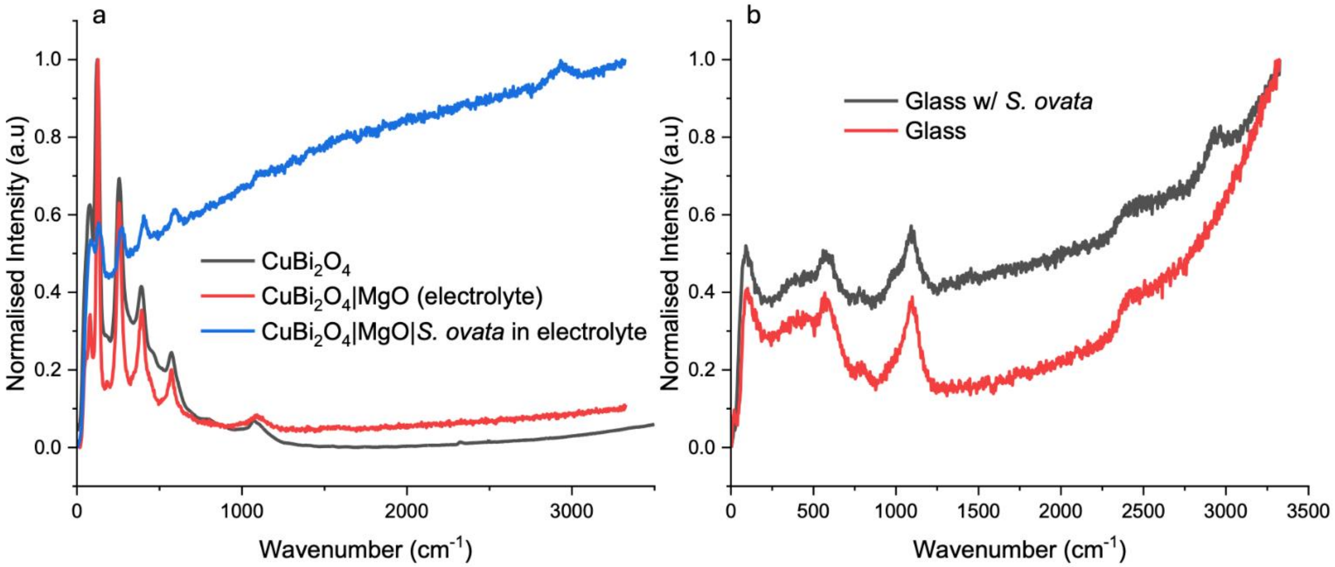


**Figure S14 – Raman spectra of $CuBi_2O_4$-based biohybrids and *Sporomusa ovata* functionalization.** ***(a)*** Raman spectra of $CuBi_2O_4$ (black), $CuBi_2O_4$|MgO in electrolyte (red), and $CuBi_2O_4$|MgO|*S. ovata* in electrolyte (blue). ***(b)*** Raman spectra of a glass substrate (red) and a glass substrate functionalized with *S. ovata* (black)

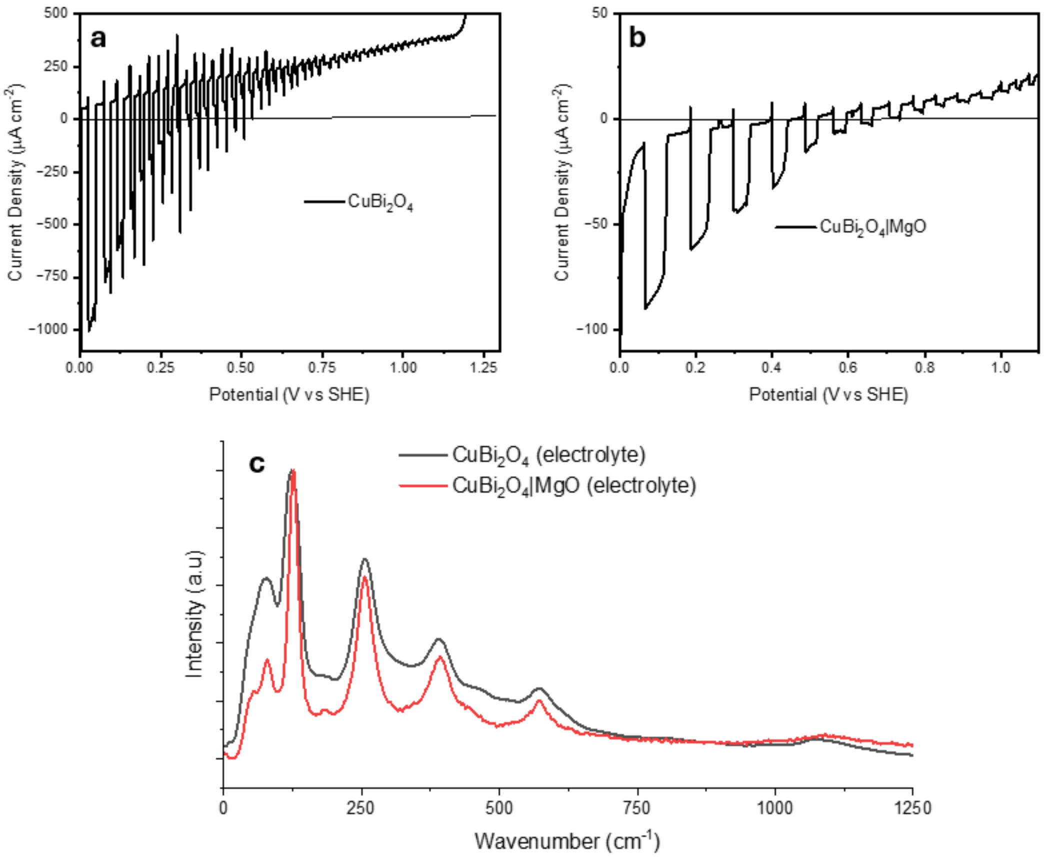


**Figure S15. (a)** Linear sweep voltammetry (LSV) of $CuBi_2O_4$ and **(b)** $CuBi_2O_4$|MgO electrodes measured versus the standard hydrogen electrode (SHE). **(c)** Raman spectra of $CuBi_2O_4$ and $CuBi_2O_4$|MgO electrodes in electrolyte. The electrolyte used for these experiments was the adapted *S. ovata* medium, as used for all electrochemical measurements throughout this study.

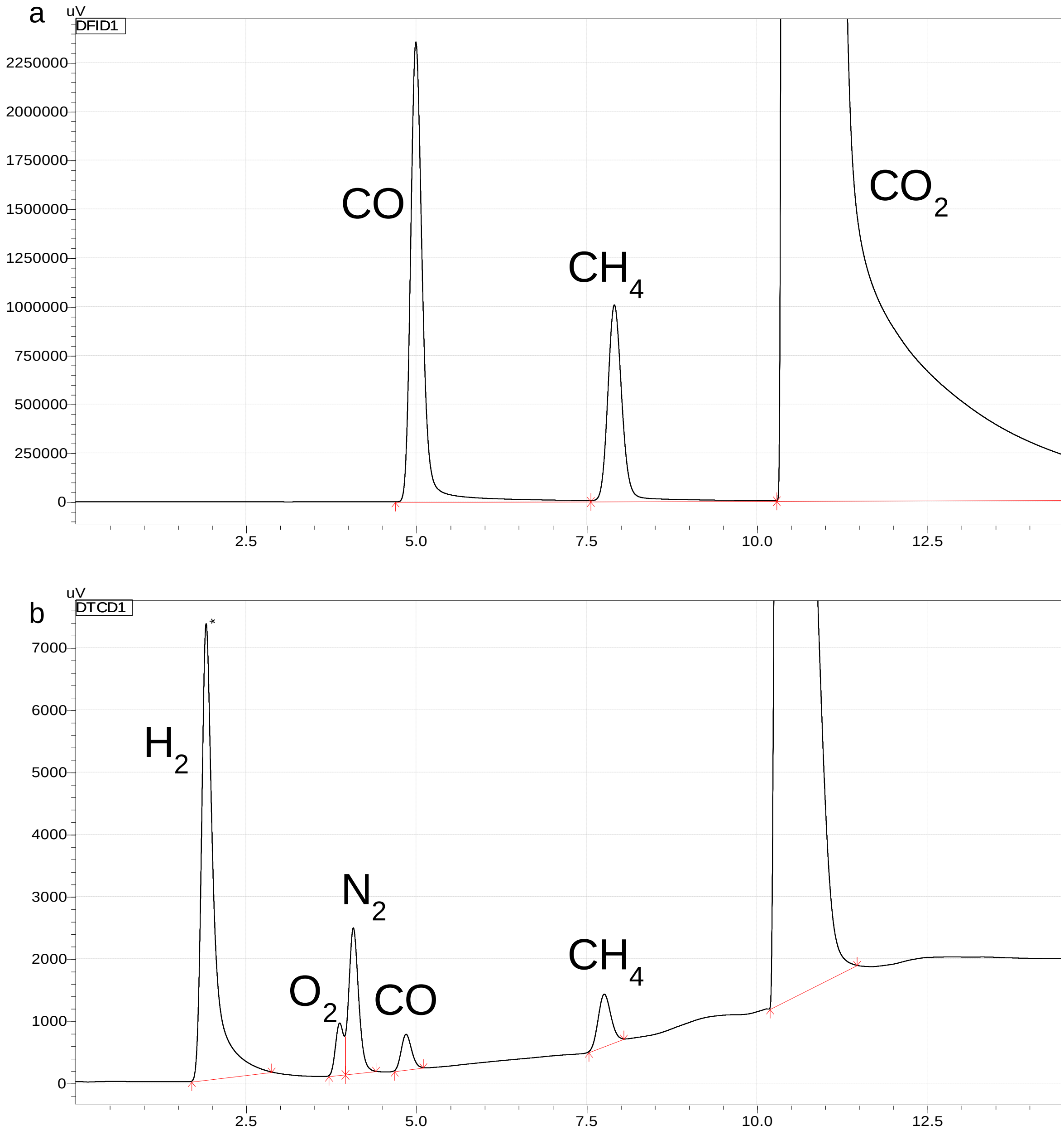


**Figure S16**. Representative GC traces of a standard gas mixture. **a)** A trace from the flame-ionization detector (FID). **b)** The trace from the thermal conductivity detector (TCD). The components of the mixture have been labelled and identified through comparing with specifications outlined by the manufacturer of the column (Restek ShinCarbon ST 80/100).

## Supporting Tables

**Table S1 –** The % abundance of different elements of the surface of $CuBi_2O_4$|MgO electrodes

| **Element** | **Wt (%)** |
|---|---|
| C | 10.74 |
| O | 12.68 |
| Bi | 14.36 |
| Cu | 59.46 |
| Ca | 1.96 |
| P | 0.79 |
| Total | 100.0 |

**Table S2 –** Medium for *Sporomusa ovata* culturing and photocatalytic reactions (DSMZ 311)

| | |
|---|---|
| $NH_4Cl$ (Sigma Aldrich, 99.998% trace metals basis) | 0.50 g |
| $MgSO_4 \cdot 7\ H_2O$ (Sigma Aldrich, BioUltra, ≥99.5%) | 0.50 g |
| $CaCl_2 \cdot 2\ H_2O$ (Sigma Aldrich, ≥99%) | 0.25 g |
| NaCl (Fisher, ≥99%) 2.25 g | 2.25 g |
| $FeSO_4 \cdot 7\ H_2O$ (Sigma Aldrich, ≥99%) (0.1% w/v in 0.1 N $H_2SO_4$ (96%)) | 2.00 mL |
| Trace element solution SL-10 (see below) | 1.00 mL |
| Selenite-tungstate solution (see below) | 1.00 mL |

| | |
|---|---|
| Yeast extract (Fisher) (omitted for photo-experiments) | 2.00 g |
| Betaine • $H_2O$ (Sigma Aldrich, ≥99%) (omitted for photo-experiments) | 6.70 g |
| $K_2HPO_4$ (Sigma Aldrich, ≥98%) | 0.35 g |
| $KH_2PO_4$ (Sigma Aldrich, ≥99%) | 0.23 g |
| $NaHCO_3$ (Fisher Scientific, 99.7-100.3%) | 4.00 g |
| Vitamin solution (see below) | 10.00 mL |
| L-Cysteine-HCl • $H_2O$ (Sigma Aldrich, ≥98%) (omitted for photo-experiments) | 0.30 g |
| MilliQ water | 1000 mL |

**Table S3 –** Product analysis from the hybrid systems with control experiments after 140-hour reaction time

| | Components | | Products (μmol $cm^{-2}$) | | |
|---|---|---|---|---|---|
| | Applied Potential (V vs SHE) | Light | [c] $H_2$ | [c] $CH_3COOH$ | [c] $CH_3CH_2OH$ |
| **$CuBi_2O_4$\|MgO** | 0 | ● | n.d. | n.d. | n.d. |
| **$CuBi_2O_4$\|MgO** | 0 | ○ | 0.076 ±0.012 | n.d. | n.d. |
| ***S. ovata*** | - | ○ | n.d. | n.d. | n.d. |
| ***S.ovata*\|$CuBi_2O_4$\|MgO[a]** | 0 | ● | n.d. | n.d. | n.d. |
| ***S.ovata*\|$CuBi_2O_4$\|MgO** | 0 | ● | n.d. | n.d. | n.d. |
| ***S.ovata*\|$CuBi_2O_4$\|MgO** | 0 | ○ | 0.066 ±0.02 | 16.830±1.79 | 17.08±1.38 |
| ***S.ovata*\|$CuBi_2O_4$\|MgO (heat-killed)[b]** | 0 | ○ | 0.078 ±0.016 | n.d. | n.d. |

[a] Without $HCO_3^-$ and purged with 100 % $N_2$. Standard error was calculated using the standard deviation (n = 3). ● = dark, ○ = illumination with 300W Xe arc lamp (1 sun, AM1.5G). n.d. = not detected. [b] *S. ovata* cells were heat-killed via autoclaving at 120 °C and 15 psi for 20 minutes. All PEC experiments were carried out in 3-neck round-

bottomed flasks with Ag/AgCl reference electrodes and Pt wires as counter electrodes. [c] Values after 140 hours of PEC.

**Table S4 |** Faradaic Efficiency calculations for acetate and ethanol concentrations after 140 hours of BPEC under constant illumination (300 W Xe lamp, 1 sun AM1.5G) at 0 V vs SHE

| Sample | Applied V vs SHE | pH | Charge passed (C) | Electrons passed (mol) | Acetate conc (M) | Ethanol conc (M) | [Acetate] / mol | [Ethanol] / mol | Faradaic efficiency Ethanol (%) | Faradaic efficiency Acetate (%) |
|---|---|---|---|---|---|---|---|---|---|---|
| 24 hrs - Acetate | 0 | 7 | 1.728 | 2.23E-06 | 17.54E-06 | - | 4.385E-07 | - | - | 19.6 |
| 24 hrs - Ethanol | 0 | 7 | 1.728 | 1.49E-06 | - | 41.4E-06 | - | 1.04E-06 | 69.4 | - |
| 48 hrs- Acetate | 0 | 7 | 3.456 | 4.47E-06 | 38.6E-06 | | 9.647E-07 | - | 0 | 21.5 |
| 48hrs- Ethanol | 0 | 7 | 3.456 | 2.98E-06 | - | 49.68E-06 | - | 1.24E-06 | 41.6 | - |
| 65hrs- Acetate | 0 | 7 | 4.68 | 6.06E-06 | 52.62E-06 | - | 1.32E-06 | - | - | 21.7 |
| 65hrs- Ethanol | 0 | 7 | 4.68 | 4.04E-06 | - | 57.96E-06 | 0 | 1.45E-06 | 35.9 | - |
| 140hrs- Acetate | 0 | 7 | 10.08 | 1.31E-05 | 175.4E-06 | - | 4.39E-06 | - | - | 33.6 |
| 140hrs- Ethanol | 0 | 7 | 10.08 | 8.70E-06 | - | 107.64E-06 | 0 | 2.69E-06 | 30.9 | - |

**Table S5 |** Medium for *Clostridium kluyveri* culturing and post-PEC fermentation (DSMZ 556)

| | |
|---|---|
| K-acetate | 10.00 g |
| $K_2HPO_4$ | 0.31 g |
| $KH_2PO_4$ | 0.23 g |
| $NH_4Cl$ | 0.25 g |
| $MgSO_4$ x 7 $H_2O$ | 0.20 g |
| Trace element solution SL-10 | 1.00 mL |
| Selenite-tungstate solution | 1.00 mL |
| Yeast extract | 1.00 g |

| | |
|---|---|
| Ethanol absolute | 20.00 mL |
| $Na_2CO_3$ | 1.00 g |
| Wolins vitamins solution | 1.00 mL |
| L-Cysteine HCl x $H_2O$ | 0.25 g |
| Distilled water | 1000.00 mL |

**Table S6 |** Trace metal solution SL-10

| | |
|---|---|
| HCl (25%; 7.7 M) | 10.00 mL |
| $FeCl_2$ • 4 $H_2O$ (Sigma Aldrich, ≥99%, trace metal basis) | 1.50 g |
| $ZnCl_2$ (Sigma Aldrich, ≥98%) | 70.00 mg |
| $MnCl_2$ • 4 $H_2O$ (Sigma Aldrich, ≥99%) | 100.00 mg |
| $H_3BO_3$ (Sigma Aldrich, ≥99.50 %) | 6.00 mg |
| $CoCl_2$ • 6 $H_2O$ (Sigma Aldrich, ≥98 %) | 190.00 mg |
| $CuCl_2$ • 2 $H_2O$ (Sigma Aldrich, ≥99.999 %) | 2.00 mg |
| $NiCl_2$ • 6 $H_2O$ (Sigma Aldrich, ≥97 %) | 24.00 mg |
| $Na_2MoO_4$ • 2 $H_2O$ (Sigma Aldrich, ≥99 %) | 36.00 mg |
| MilliQ water | 990.00 mL |

**Table S7 |** Selenite-tungstate solution

| | |
|---|---|
| NaOH (Sigma Aldrich, ≥98 %) | 0.50 g |
| $Na_2SeO_3$ • 5 $H_2O$ (Sigma Aldrich, 98 %) | 3.00 mg |

| | |
|---|---|
| $Na_2WO_4 \cdot 2 H_2O$ (Sigma Aldrich, ≥99%) | 4.00 mg |
| MilliQ water | 1000.00 mL |

**Table S8 |** Vitamin solution

| | |
|---|---|
| Biotin (Sigma Aldrich, ≥99%) | 2.00 mg |
| Folic acid (Sigma Aldrich, ≥97%) | 2.00 mg |
| Pyridoxine-HCl (Sigma Aldrich, ≥98%) | 10.00 mg |
| Thiamine-HCl $\cdot$ 2 $H_2O$ (Sigma Aldrich, ≥99%) | 5.00 mg |
| Riboflavin (Fisher Scientific, Across Organics, 98%) | 5.00 mg |
| Nicotinic acid (Sigma Aldrich, ≥98%) | 5.00 mg |
| D-Ca-pantothenate (Sigma Aldrich, ≥98%) | 5.00 mg |
| Vitamin B12 (Sigma Aldrich, ≥98%) | 0.10 mg |
| p-Aminobenzoic acid (Sigma Aldrich, ≥99%) | 5.00 mg |
| Lipoic acid (Sigma Aldrich, ≥98%) | 5.00 mg |
| $H_3BO_3$ (Sigma Aldrich, ≥99.50%) | 0.01 g |
| MilliQ water | 1000.00 mL |

**Table S9 |** Comparative summary of reported *Sporomusa ovata*–based biohybrid systems. The table lists semiconductor–microbe assemblies, operating conditions (illumination, mediators, atmosphere), product performance (acetate and/or ethanol titers, yields, or efficiencies), stability (operating duration), and literature references.

| **System** | **Conditions** | **Performance** | **Stability** | **Reference** |
|---|---|---|---|---|
| **$CuBi_2O_4$\|MgO\|*S. ovata*** | No mediators, no SED. 1 sun illumination, Xe lamp, AM1.5G (100mW $cm^{-2}$) | 673.2±71.4 μM $cm^{-2}$ of acetate and 683.1±55.2 μM $cm^{-2}$ of ethanol. | 5.5+ days | This work |
| **SiNW\|*S. ovata*** | Simulated sunlight (AM1.5G, 100 mW $cm^{-2}$); purged with 20% $CO_2$ / 80% $N_2$; neutral pH; no mediators/SED; $O_2$-tolerant operation also shown (21% $O_2$/10% $CO_2$/69% $N_2$). | Faradaic efficiency up to 90% for acetate; ~6 g $L^{-1}$ acetate after 5 days. | Continuous operation >5 days; stability reported up to 200 h. | [1] |
| **$TiO_2$\|SiNW\|*S. ovata*** | $TiO_2$ nanowire photoanode (water oxidation) wired to Si nanowire photocathode ($H_2$ evolution feeding S. ovata); unassisted under simulated sunlight (AM1.5G, 100 mW $cm^{-2}$). | Same device as above used for unassisted $CO_2$→acetate; FE up to 90%; ~6 g $L^{-1}$ acetate in 5 days. | >5 days operation demonstrated; up to 200 h stability. | [2] |
| **SrTiO3:La,Rh\|\| BiVO4:Mo\|*S. ovata*** | Particulate “photocatalyst sheet” Z-scheme, no mediators/SED; 1 sun AM1.5G (100 mW $cm^{-2}$); $CO_2$-purged medium | ~9 mM acetate in 15 h; solar-to-acetate = 0.70 ± 0.04 %; ~90% selectivity | Operated in 3 × 15 h runs ($Cr_2O_3$ reloaded) with ~82% activity retained after 45 h total. | [3] |

| | | | | |
|---|---|---|---|---|
| | (5–20% $CO_2$), pH ~7.2 | | | |
| **ITO/ZnO/P3HT:PCBM/ $MoO_3$** | Simulated sunlight for 12 hours and darkness for 12 hours at 34°Cunder N 2/CO2 (80/20). cysteine and potassium ferricyanide were added every 24 hours as hole-trapping agents and electron mediators, | Acetate yield of 8.2 g / week was obtained with a 400-$cm^2$ area | 35+ days | [4] |
| **CdS\|*S. ovata*** | LED array lamp (400 ± 5 nm) at 0.20 $mW/cm^2$ (low intensity) or 2.00 $mW/cm^2$ (high intensity) under 30 °C with magnet stirring at 1000 rpm. Cysteine as SED. | ~22 mM Acetate in 6 days | 7 days | [5] |
| **$Cd_{0.8}Zn_{0.2}S$\|*S. ovata*** | LED array composed of violet LEDs (450 ± 5 nm) for blue light measurements at a light intensity of $20Wm^{-2}$. Magnetically stirred (120 rpm) at 30 ºC | ~50 mM Acetate in 6.5 days | 6.5 days | [6] |

## Supporting Equations

$$\frac{P}{V(P_0 - P)} = \frac{1}{V_m C} + \frac{(C-1)P}{V_m C P_0} \qquad \text{(S1)}$$

$$J_{DL} = C_{DL} \times \frac{v}{A} = 1.228 \times 10^{-5}\ F \quad (S2)$$

$$ECSA = \frac{C_{DL}}{C_e} = \frac{1.228 \times 10^{-5} F}{40 \times 10^{-6}\ Fcm^{-2}} \quad (S3)$$


**Supporting References**

1. Liu, C. *et al.* Nanowire–bacteria hybrids for unassisted solar carbon dioxide fixation to value-added chemicals. *Nano Lett.,* **2015**, 15, 3634-3639.
2. Su, Y. *et al.* Close-Packed Nanowire-Bacteria Hybrids for Efficient Solar-Driven CO2 Fixation. *Joule* , **2020**, 4, 800-811. https://doi.org/10.1016/j.joule.2020.03.001
3. Wang, Q. *et al*. Bacteria–photocatalyst sheet for sustainable carbon dioxide utilization. *Nat Catalysis,* **2022**, 5, 633-64. https://doi.org/10.1038/s41929-022-00817-z
4. Wen, N. *et al*. Polymer semiconductor films and bacteria hybrid artificial bio-leaves, *Sci. Adv.,* **2024**, 10, 44.
5. Ying, H.*et al.*, Photosynthesis of Acetate by *Sporomusa ovata*-CdS Biohybrid System, *ACS Appl, Mater. Interfaces,* ***2022**, 14, 20, 23364-23374*
6. Zhang, K. *et al.,* Biohybrids of twinning Cd0.8Zn0.2S nanoparticles and *Sporomusa ovata* for efficient solar-driven reduction of $CO_2$ to acetate, Appl. Catal. B - Environ., **2024**, 324,123375.
